# Epigenetic DNA Modifications Are Correlated With B Chromosomes and Sex in the Cichlid *Astatotilapia latifasciata*

**DOI:** 10.3389/fgene.2019.00324

**Published:** 2019-04-12

**Authors:** Adauto Lima Cardoso, Bruno Evaristo de Almeida Fantinatti, Natália Bortholazzi Venturelli, Bianca de Oliveira Carmello, Rogério Antonio de Oliveira, Cesar Martins

**Affiliations:** ^1^Integrative Genomics Laboratory, Department of Morphology, Institute of Biosciences, São Paulo State University – Universidade Estadual Paulista, Botucatu, Brazil; ^2^Department of Biostatistics, Institute of Biosciences, São Paulo State University – Universidade Estadual Paulista, Botucatu, Brazil

**Keywords:** supernumerary chromosome, 5-methylcytosine, 5-hydroxymethylcytosine, DNA methylation, DNA demethylation, microRNA

## Abstract

Supernumerary B chromosomes are dispensable elements found in several groups of eukaryotes, and their impacts in host organisms are not clear. The cichlid fish *Astatotilapia latifasciata* presents one or two large metacentric B chromosomes. These elements affect the transcription of several classes of RNAs. Here, we evaluated the epigenetic DNA modification status of B chromosomes using immunocytogenetics and assessed the impact of B chromosome presence on the global contents of 5-methylcytosine (5mC) and 5-hydroxymethylcytosine (5hmC) and the molecular mechanisms underlying these variations. We found that the B chromosome of *A. latifasciata* has an active pattern of DNA epimarks, and its presence promotes the loss of 5mC in gonads of females with B chromosome (FB+) and promotes the loss of 5hmC in the muscle of males with the B element (MB+). Based on the transcriptional quantification of DNA modification genes (*dnmt, tet*, and *tdg*) and their candidate regulators (*idh* genes, microRNAs, and long non-coding RNAs) and on RNA-protein interaction prediction, we suggest the occurrence of passive demethylation in gonads of FB+ and 5hmC loss by Tet inhibition or by 5hmC oxidation in MB+ muscle. We suggest that these results can also explain the previously reported variations in the transcription levels of several classes of RNA depending on B chromosome presence. The DNA modifications detected here are also influenced by sex. Although the correlation between B chromosomes and sex has been previously reported, it remains unexplained. The B chromosome of *A. latifasciata* seems to be active and impacts cell physiology in a very complex way, including at the epigenetic level.

## Introduction

Supernumerary B chromosomes (B) are numerical chromosome polymorphisms reported in several groups of eukaryotes, including plants, fungi and animals (Jones, 2017). B chromosomes are dispensable, exhibit non-Mendelian patterns of inheritance and were traditionally seen as inert elements (Beukeboom, 1994; Camacho et al., 2000). However, the identification of functional sequences in B chromosomes and the effects of these elements in hosts changed the view of these chromosomes as non-functional units (Camacho et al., 2000; Banaei-Moghaddam et al., 2015). Moreover, recent advances based on large-scale DNA/RNA analyses have allowed an understanding of B chromosome biology at a level never considered before (Martis et al., 2012; Valente et al., 2014, 2017; Huang et al., 2016; Li et al., 2017; Ruban et al., 2017; Navarro-Domínguez et al., 2019). This new scenario has provided evidence of complex B chromosome effects in the cells and organisms. In several species B chromosomes have sex-associated differences in frequency, effects and transmission, and they can even generate or were derived from sex chromosomes (Camacho et al., 2011). The influence of Bs over sex seems to be one of the most fascinating and still not understood phenotypic effects of the presence of extra chromosomal elements.

The African cichlid *Astatotilapia latifasciata* has a standard karyotype with 44 chromosomes and supernumerary chromosomes that can be found in several individuals, ranging from 0 to 2 (Poletto et al., 2010; Fantinatti et al., 2011). This B chromosome is large, metacentric, fully heterochromatic and rich in repetitive sequences (Poletto et al., 2010; Fantinatti et al., 2011; Valente et al., 2014). Among the repetitive DNA classes, a sequence that is enriched in the B chromosome and called BncDNA was characterized as a potentially long non-coding RNA and identified as differentially processed and differentially expressed by the effect of supernumerary chromosomes (Ramos et al., 2017). Expansion of several transposable elements in the B chromosome was found (Coan and Martins, 2018). The B chromosome carries potentially duplicated protein-coding genes (Valente et al., 2014) and retro-inserted hnRNP Q-like genes (Carmello et al., 2017). To the best of our knowledge, the B chromosome presence seems to impact the transcription levels of several classes of RNA, thereby influencing cellular function (Carmello et al., 2017; Ramos et al., 2017; Valente et al., 2017; Coan and Martins, 2018).

Despite the reported functional effects of the B chromosome in *A. latifasciata* cells, it is still not clear how the B element has such an influence. Moreover, total or partial inactivation of this element should be necessary to avoid gene dosage effects, such as those observed in aneuploidies (Han et al., 2007). A wide range of regulatory mechanisms may be involved in these processes, and the epigenetic regulation of chromatin seems to be one of possible pathways that B chromosome uses to impact cell physiology, since chromatin modifications directly impact the regulation of gene expression (Cedar and Bergman, 2009). Among these modifications, DNA methylation is described as inactivating complete B chromosomes (Bugno-Poniewierska et al., 2014) or specific B-sequences (López-León et al., 1991; Leach et al., 1995; Langdon et al., 2000; Stitou et al., 2000; Koo et al., 2011) and can be a mechanism that silences transposable elements in the B chromosome of *A. latifasciata* (Coan and Martins, 2018). In turn, the presence of a B chromosome can affect the DNA methylation status of the cell and thereby impact the global profile of gene expression, as observed in aneuploidies (Mendioroz et al., 2015), another type of numerical variation.

DNA methylation (5mC formation) is carried out by the enzyme class DNA methyltransferases (Dnmt1, Dnmt3a, and Dnmt3b) (Plongthongkum et al., 2014). These enzymes catalyze the addition of methyl radicals on cytosines. Dnmt3a and Dnmt3b have *de novo* methylation function, and Dnmt1 is the maintainer Dnmt (Goll and Bestor, 2005). Although DNA methylation is well studied, active and passive DNA demethylation (5mC removal) is poorly explored. Active DNA demethylation is conducted by dioxygenases of the ten-eleven translocation family (Tet1, Tet2, and Tet3) and thymine DNA glycosylase (Tdg), which promote oxidation and base excision, respectively (Kohli and Zhang, 2013). This process involves the transient formation of the variants 5-hydroxymethylcytosine (5hmC), 5-formylcytosine (5fC), and 5-carboxylcytosine (5caC), from 5mC until the recovery of the unmethylated cytosine status (Ito et al., 2011; Kohli and Zhang, 2013; Shen et al., 2014). In turn, passive DNA demethylation occurs during DNA replication in the absence of the maintainer Dnmt activity (Kohli and Zhang, 2013). In fish, the percentage of DNA methylation in CpG contexts is usually higher than in mammal and bird species (Varriale and Bernardi, 2006; Goll and Halpern, 2011; Head, 2014), which suggests the relevance of this epigenetic change to genome function. Epigenetic DNA modifications can be assessed by several methods reaching from cytological approaches, as chromosome immunostaining (Rens et al., 2010; Costa et al., 2016), to strategies that provide the epigenetic status of cytosines (Harrison and Parle-McDermott, 2011; Kurdyukov and Bullock, 2016).

Deregulation of Dnmt, Tet, and Tdg activity can change the pattern of DNA epimarks, and this can result in disturbances of the transcriptional or posttranscriptional mechanisms that regulate the expression of the *dnmt, tet*, and *tdg* genes (Denis et al., 2011; Arvinden et al., 2017). Among these mechanisms, the action of microRNAs (miRNA) is widely recognized (Kuc et al., 2017). miRNAs are a class of short (∼20–22 nucleotides), non-coding RNAs that regulate gene expression by target 3′UTR regions of mRNAs (Bartel, 2004) in normal and pathological conditions (Alvarez-Garcia and Miska, 2005). Protein–RNA interactions are a regulatory mechanism of DNA modification enzymes (Di Ruscio et al., 2013). Deregulated activity of isocitrate dehydrogenase enzymes (Idh1 and Idh2), which catalyze the conversion of isocitrate to α-ketoglutarate, a cofactor to Tet, can affect the functions of these dioxygenases and DNA demethylation (Figueroa et al., 2010; Turcan et al., 2012).

Considering that B chromosomes of *A. latifasciata* are the source of several classes of RNAs (Valente et al., 2014, 2017; Carmello et al., 2017; Ramos et al., 2017; Coan and Martins, 2018), it is possible these chromosomal elements regulate DNA modification genes in several ways and promote epigenetic effects on a DNA level. Therefore, to understand if the B chromosome promotes epigenetic DNA modifications in the host cells, we analyzed the DNA modification status of this chromosome and its effects on the routes of DNA methylation and demethylation. In terms of DNA epimarks, the B chromosome of *A. latifasciata* seems to be active and impacts heterogeneously the levels of 5mC and 5hmC, which can in part explain the differential expression of several classes of RNAs induced by B chromosomes.

## Materials and Methods

### Samples

Twenty-nine mature *A. latifasciata* individuals [5 males without B (MB-), 10 males with B (MB+), 4 females without B (FB-), and 10 females with (FB+)], which constituted the offspring of two crosses between MB+ and FB+ individuals maintained in standardized environmental conditions in the fish facility of Integrative Genomics Laboratory at São Paulo State University (UNESP), Botucatu, Brazil, were used in this study. The first cohort was 180 days old and was composed of 3 MB-, 6 MB+, 3 FB-, and 5 FB+. The second cohort was 210 days old and was composed of 2 MB-, 4 MB+, 1 FB-, and 5 FB+. All the procedures were in accordance with the ethical principles of the Brazilian College of Animal Experimentation and were approved by the Institute of Biosciences (UNESP) ethics committee on the use of animals (Protocol No. 486-2013; 769-2015). The individuals were genotyped for B chromosome presence by PCR with a set of primers developed by Fantinatti and Martins (2016). The PCR protocol only detected the B chromosome presence and did not allow discrimination of 1 or 2 B chromosomes. Therefore, B+ samples included 1 or 2 B chromosomes.

### DNA and RNA Extraction

Total DNA was obtained from the caudal fin for genotyping (B chromosome absence or presence) and from the encephalon, muscle and gonad for 5mC and 5hmC global quantification. Previous gene ontology analysis of B chromosome gene copies (Valente et al., 2014) revealed enrichment of terms related to these tissues and, therefore, they were selected for the present study. DNA was extracted with phenol-chloroform (Sambrook and Russel, 2001), and its integrity was assessed using agarose gel electrophoresis. Total RNA was extracted from 10 mg of encephalon, muscle and gonads with TRIzol^®^ Reagent (Thermo Fisher Scientific, cat. 15596026) following the manufacturer’s instructions. RNA integrity number (RIN) was assessed in a Bioanalyzer 2011, and only samples with RIN greater than or equal to 7 and 8 were used in the Quantitative Reverse Transcription PCR (RT-qPCR) experiments and small RNA sequencing, respectively. Nucleic acid concentrations were determined with a Nanodrop 2000 spectrophotometer.

### Chromosome Immunostaining

Metaphasic chromosomes used in the immunostaining of DNA modifications were obtained from kidney cells, which constitute the hematopoietic tissue of fish, following the protocol of Bertollo et al. (1978). Chromosomes were fixed in Carnoy solution (3:1 methanol:acetic acid). Immunofluorescence with 5mC (Abcam, cat. Ab-124936) and 5hmC antibodies (Active Motif, cat. 39770) was performed following the protocol of Rens et al. (2010) with modifications. Cell suspensions containing the metaphasic chromosomes were dropped on slides, followed by dehydration in an ethanol series (70, 85, and 100%). The slides were denatured in 2 N HCl for 15 min at 37°C, incubated in 0.1 M borax (pH 8.4) for 1 min, incubated in PBS for 1 min, dehydrated in an ethanol series and rehydrated in PBS for 3 min. The slides were covered with 10% bovine serum for 7 min, followed by treatment with a primary antibody overnight at 4°C. The slides were washed three times for 5 min each in PBS-Tween 0.01%. The slides were then covered with a 1:100 ratio of secondary antibody anti-rabbit IgG-FITC (Sigma-Aldrich, cat. F0382) in PBS-Tween 0.01% for 40 min and washed as before. Finally, the slides were mounted in Vectashield with DAPI (Vector Laboratories, cat. H-1200). Line scans of the chromosomes were obtained using ImageJ software to determine the levels of fluorescence along each chromosome. Thirty cells of each three individuals (one male and two female) with one B chromosome were analyzed to detect 5mC and 5hmC signal distribution and levels of fluorescence. One metaphase was selected to represent the results of immunostaining and line scan.

### Global DNA Methylation and Hydroxymethylation

The global DNA methylation (5mC) and hydroxymethylation (5hmC) were quantified in three individuals of each group (MB-, MB+, FB-, and FB+) with the colorimetric MethylFlash Methylated DNA Quantification and MethylFlash Hydroxymethylated DNA Quantification kits (Epigentek, cat. P-1034, P-1036), respectively, following the manufacturer’s instructions. The experiments were performed in technical duplicates with an initial amount of DNA of 100 ng per sample. The relative percentages of methylated and hydroxymethylated DNA in relation to positive control were detected by measuring the absorbance at 450 nm and using the formula described by the manufacturer: 5mC% or 5hmC% = ((Sample OD – NC OD)/S × 100%)/((PC OD – NC OD) × F^∗^)/R, where OD is optical density; NC is negative control (a unmethylated polynucleotide containing 50% of cytosine used for 5mC quantification or a methylated polynucleotide containing 20% of 5-methylcytosine used for 5hmC quantification) supplied by the manufacturer; PC is positive control (a methylated polynucleotide containing 50% of 5-methylcytosine used for 5mC quantification or a hydroxymethylated polynucleotide containing 20% of hydroxymethylcytosine used for 5hm quantification) supplied by the manufacturer; S is amount of input sample DNA in nanograms; F is a factor to normalize 5mC or 5hmC in the positive control to 100%, as the positive control contained only 50% of 5mC or 20% of 5hmC, where *F* = 2 for 5mC and *F* = 5 for 5hmC quantification; and R, amount of input positive control in nanograms.

### miRNA Target Prediction

To determine if miRNAs regulate DNA modification genes, the 3′UTRs of these genes were obtained in the gene annotations available on SaciBase^1^ and BouillaBase^2^. Sequences of the expressed miRNAs were acquired by alignments of the reads of the small RNA sequenced libraries (Fantinatti, 2015) against the fish mature miRNA sequences downloaded from miRBase v.21^3^. MicroRNA target predictions were performed using PITA (Kertesz et al., 2007), miRanda (Enright et al., 2004) and RNAhybrid (Krüger and Rehmsmeier, 2006). Overlapping interactions in the three predictors with *p*-value less than 0.05 and free energy less than -10 kcal/mol for PITA and -18 kcal/mol for miRanda and RNAhybrid were considered significant.

### miRNA Identification and Expression

Small RNA sequencing of two libraries of muscle and three of encephalon and gonads of both groups were performed using single-end Illumina HiSeq 2000 platform. The quality of sequencing data was analyzed with FastQC software, and reads with low quality were eliminated with FastX-toolkit following quality thresholds: 90% of read extension with a phred score of at least 30. Mature miRNA was identified by alignment of filtered reads against a mature miRNA sequence dataset of fish downloaded from miRBase v.21 using Bowtie2 software. The alignments did not accept mismatches and all the parameters were kept on default. Only the miRNAs that were found as candidates to regulate DNA modification genes had their expression determined. Count data normalization and differential expression (DE) of mature miRNAs were carried out using R/Bioconductor DESeq1 package (Anders and Huber, 2010) that is based on the negative binomial distribution with variance and mean linked by local regression. No filtering after read count obtention was carried out. We considered DE miRNAs those that showed fold-change ≥2 and *p*-value ≤ 0.05.

### mRNA and miRNA Expression Validation

Relative expression of mRNA and miRNA was assessed in 5 MB-, 9 MB+, 4 FB-, and 8 FB+. RNA was treated with DNase I, RNase-free Kit 1 U/μL (Thermo Fisher Scientific, cat. EN0521). Reverse transcription (RT) of mRNA was performed using a High Capacity kit (Thermo Fisher Scientific, cat. 4368814) with an initial 1,000 ng of RNA. RT of miRNA was conducted in agreement with Mei et al. (2012), which included a first step of miRNA polyuridylation with the initial 1000 ng of RNA and cDNA synthesis with a poly(A)-stem-loop primer. For quantification based on qPCR, we used 4 ng of cDNA amplified with GoTaq qPCR Master Mix kit (Promega, cat. A6001) and 400 nM of each primer in a final volume of 20 μL. U6 snRNA and *ubce* genes were used as a reference for normalization of the miRNA and mRNA expression, respectively. Both targets and reference genes were analyzed in duplicate. Cycling conditions were as follows: 95°C for 5 min and 40 cycles at 95°C for 15 s and 60°C for 1 min. The thermal cycler was a StepOne Plus Real-Time PCR System (Thermo Fisher Scientific). The ΔΔCt method was used to compare the relative gene expression. The data were normalized using Q-Gene software (Muller et al., 2002; Simon, 2003). The sequences of the primers (specific for cDNA amplification) used are available in Supplementary Table S1.

### RNA-Protein Interaction Prediction

The potential non-coding BncRNA is differentially expressed in B chromosome samples, mainly the BncRNA region 2, which is upregulated in B+ samples (Ramos et al., 2017). Moreover, non-coding RNAs were described as inhibitors of Dnmt1 (Di Ruscio et al., 2013). Thus, to explore the possible association of BncRNA and Dnmt, we performed interaction predictions of the BncRNA complete sequence and BncRNA region 2 with the Dnmts amino acid sequences using the tools RPIseq (Muppirala et al., 2011) and RPI-pred (Suresh et al., 2015).

### Statistics

The results of the global quantification of 5mC and 5hmC, mRNA and miRNA transcription were analyzed as in Carmello et al. (2017) and Ramos et al. (2017) using a generalized linear model (GLM) statistical approach with averages adjusted by the asymmetric gamma distribution of all the variables, implemented in SAS version 9.3, procedure GENMOD, followed by Bonferroni’s *post hoc* test. Associations of 5mC level and BncRNA expression were determined by Pearson’s correlation. In all the cases, significant results were accepted at a maximum *p*-value of 0.05.

## Results

### Chromosomal Immunolocalization of 5mC and 5hmC Marks

**FIGURE 1:**
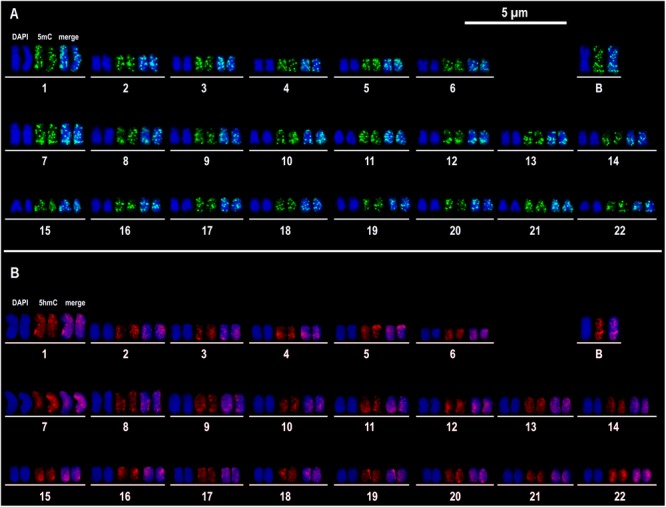
Immunocytogenetics in *Astatotilapia latifasciata*. Representative image of immunostaining of 5mC **(A)** and 5hmC **(B)** in the metaphasic chromosomes of one cell. 5mC and 5hmC signals are observed in green and red, respectively. The chromosomes are counterstained in blue with DAPI. For each karyotype chromosome pair, the fluorescent signals of DAPI and 5mC or 5hmC are shown separated and merged.

The chromosomal immunolocalization of 5mC showed marks scattered over all A chromosomes and on the B chromosome, without accumulation in any specific region and without differences between A and B chromosomes (Figure 1A). Similarly, the 5hmC marks were dispersed over the A and B chromosomes without accumulation in any specific region and without differences between A and B chromosomes (Figure 1B). Line scan provided a more detailed signal distribution of marks and showed peaks of 5mC marks along all the chromosomes and regions of depletion of these marks, as the centromeric regions of most chromosomes, including the centromeric region of the B chromosome (Supplementary Figure S1). In turn, line scan showed that 5hmC marks were distributed similarly along A and B chromosomes (Supplementary Figure S2). Since we studied chromosomes of one MB+ and two FB+ samples, we did not observe differences among the three individuals.

### Effects of B Chromosomes and Sex on the Global Levels of 5mC and 5hmC

**FIGURE 2:**
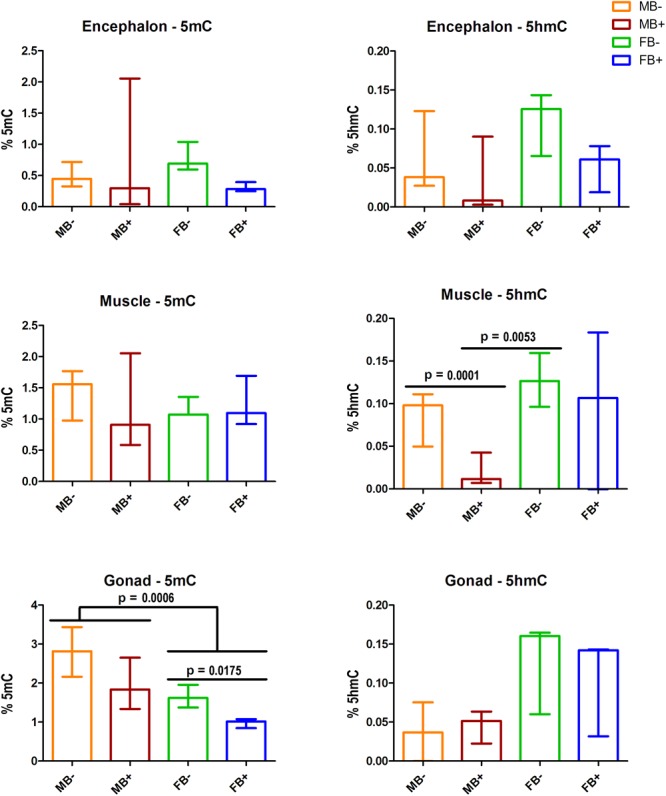
DNA modifications in the presence of B chromosome in *A. latifasciata*. Global quantification of 5mC and 5hmC in the encephalon, muscle and gonads. Statistically significant *p*-values determined by *t*-test (α = 0.05) are indicated by horizontal lines. Bar colors represent the different samples analyzed, which included males without B chromosome (orange, MB-), males with B chromosome (brown, MB+), females without B chromosome (green, FB-), and females with B chromosome (blue, FB+).

We assessed the contents of 5mC and 5hmC in encephalon, muscle and gonads of MB-, MB+, FB-, and FB+ samples (Figure 2). In encephalon, we did not find statistically significant differences in the levels of 5mC and 5hmC among the groups, although we observed a tendency of reduction of 5mC in FB+ compared to FB-. In muscle, we did not observed variations in the level of 5mC among the groups, but the level of 5hmC was different between MB- and MB+ (*p* = 0.0001; α = 0.05) and between MB+ and FB- (*p* = 0.0053; α = 0.05). In gonads, we identified statistical differences in the level of 5mC between males and females (*p* = 0.0006; α = 0.05) and between FB- and FB+ (*p* = 0.0175; α = 0.05). In addition, we observed a tendency of increase of the level of 5hmC in females, but that was not statistically significant.

### Transcription of Genes of DNA Modification Cycle

**FIGURE 3:**
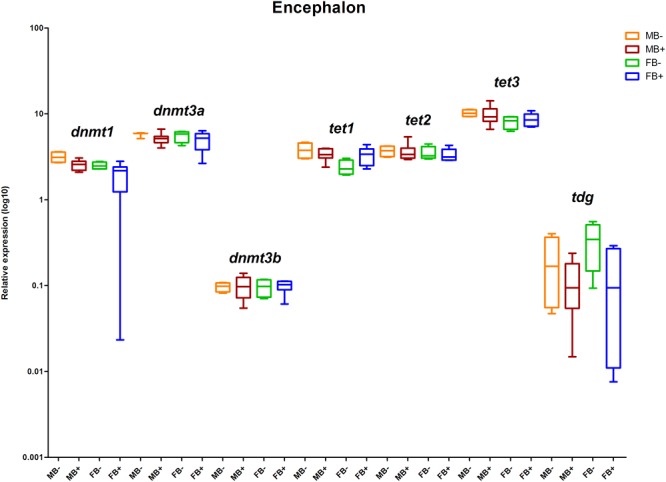
Relative expression of DNA modification genes in *A. latifasciata*. Transcription levels of the genes *dnmt1, dnmt3a, dnmt3b, tet1, tet2, tet3*, and *tdg* in encephalon measured by RT-qPCR. *p, p*-values determined by generalized linear model (α = 0.05) followed by Bonferroni’s *post hoc* test. Bar colors represent the different samples analyzed, which included males without B chromosome (orange, MB-), males with B chromosome (brown, MB+), females without B chromosome (green, FB-), and females with B chromosome (blue, FB+). The box define median and the lines the minimum and maximum values.

**FIGURE 4:**
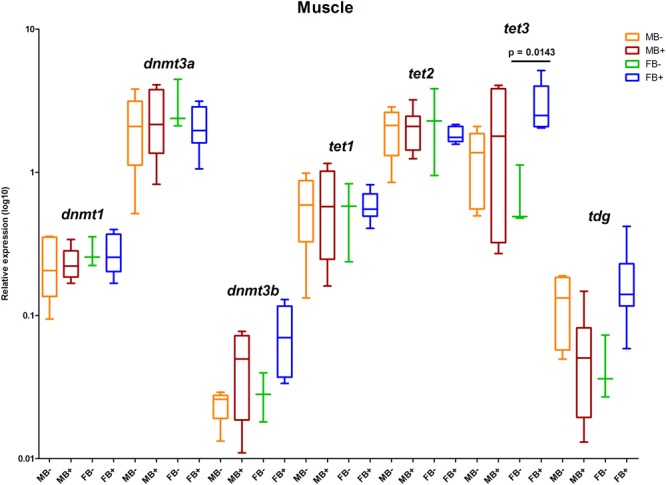
Relative expression of DNA modification genes in *A. latifasciata*. Transcription levels of the genes *dnmt1, dnmt3a, dnmt3b, tet1, tet2, tet3*, and *tdg* in muscle measured by RT-qPCR. *p*-values determined by generalized linear model (α = 0.05) followed by Bonferroni’s *post hoc* test. Bar colors represent the different samples analyzed, which included males without B chromosome (orange, MB-), males with B chromosome (brown, MB+), females without B chromosome (green, FB-), and females with B chromosome (blue, FB+). The box define median and the lines the minimum and maximum values.

**FIGURE 5:**
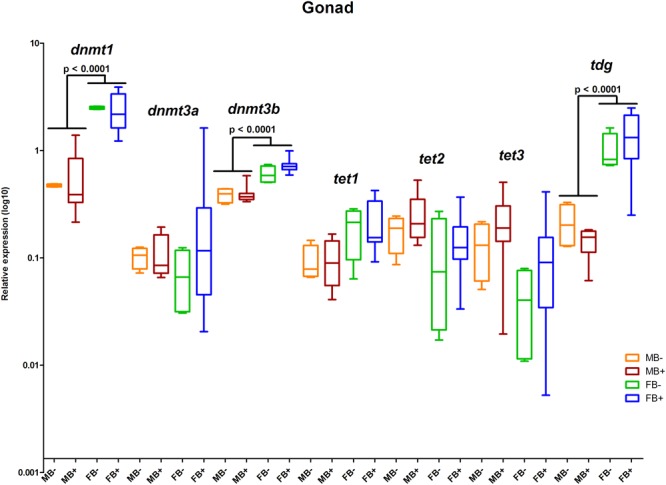
Relative expression of DNA modification genes in *A. latifasciata*. Transcription levels of the genes *dnmt1, dnmt3a, dnmt3b, tet1, tet2, tet3*, and *tdg* in gonads measured by RT-qPCR. *p, p*-values determined by generalized linear model (α = 0.05) followed by Bonferroni’s *post hoc* test. Bar colors represent the different samples analyzed, which included males without B chromosome (orange, MB-), males with B chromosome (brown, MB+), females without B chromosome (green, FB-), and females with B chromosome (blue, FB+). The box define median and the lines the minimum and maximum values.

**FIGURE 6:**
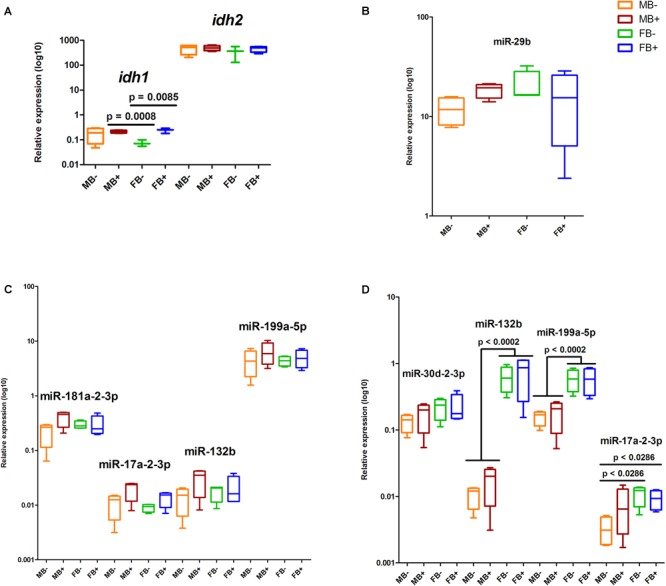
Relative expression of the regulators of DNA modification genes in *A. latifasciata*. Transcription levels of *idh* genes in the muscle measured by RT-qPCR **(A)**. Transcription levels of selected candidate miRNAs regulating the DNA modification genes in encephalon **(B)**, muscle **(C)**, and gonads **(D)**, as revealed by RT-qPCR. *p, p*-values determined by generalized linear model (α = 0.05) followed by Bonferroni’s *post hoc* test. Bar colors represent the different samples analyzed, which included males without B chromosome (orange, MB-), males with B chromosome (brown, MB+), females without B chromosome (green, FB-), and females with B chromosome (blue, FB+). The box define median and the lines the minimum and maximum values.

We determined the relative expression of DNA modification genes in encephalon, muscle and gonads of MB-, MB+, FB-, and FB+ samples. In encephalon, we did not detect statistically significant differences in the transcription of *dnmt1, dnmt3a, dnmt3b, tet1, tet2, tet3*, or *tdg* among the groups (Figure 3). In addition, we observed a tendency of reduction in the transcription of *tdg* gene in encephalon of MB+ and FB+, although these variations have not been statistically significant. In muscle, we identified significant differences only in the level of expression of the gene *tet3* between FB- and FB+ (*p* = 0.0143; α = 0.05) (Figure 4). Moreover, we found a tendency of increase of *dnmt3b* transcription in muscle of MB+ and FB+, and reduction of *tdg* trancription in MB+ and increase of *tdg* transcription in FB+, although these variations have not been statistically proven. In gonads, we detected differences in the transcription of *dnmt1, dnmt3b*, and *tdg* between males and females independent of B chromosome presence (*p* < 0.0001; α = 0.05) (Figure 5). We also found a tendency of reduction of *tet3* expression in ovaries of FB+, although this has not been statistically significant. Additionally, in muscle, we observed differences in the transcription of *idh1* between MB+ and FB- (*p* = 0.0008; α = 0.05) and between FB- and FB+ (*p* = 0.0085; α = 0.05) (Figure 6A).

### Identification and Expression of epi-miRNAs

Using alignments of mature miRNA sequences against the reads of small RNA-seq libraries (Fantinatti, 2015), we identified the set of miRNAs expressed in *A. latifasciata* (data not shown). Then, we used these identified miRNAs to predict miRNA/3′UTR interactions and found candidate miRNAs to regulate DNA modification genes in this species (Table 1). Next, we explored their expression profile in small RNA-seq libraries (Fantinatti, 2015), and in agreement with the cutoff used here, we did not found different expressed miRNAs among the groups (Supplementary Table S2). We selected some miRNAs to validate their expression by RT-qPCR and found no differences in the expression of the miR-29b in the encephalon among the groups (Figure 6B). We did not observe changes in the expression of miRNAs in muscle (Figure 6C). We found variations in the expression of miR-132b (*p* = 0.0002; α = 0.05) and miR-199a-5p (*p* = 0.0002; α = 0.05) between testicles and ovaries independent of B chromosome presence (Figure 6D). In gonads, we also identified differences in the expression of miR-17a-2-3p between MB- and FB- (*p* = 0.0286; α = 0.05) and between MB- and FB+ (*p* = 0.0286; α = 0.05).

**Table 1:** Candidate miRNAs in the regulation of DNA modification genes predicted by the software PITA, miRanda, and RNAhybrid.

Target gene	miRNA
*dnmt1*	ssa-miR-143-5p, ssa-miR-30d-2-3p
*dnmt3a*	dre-miR-29b
*dnmt3b*	ccr-miR-132b/dre-miR-29b
*tet1*	unidentified
*tet2*	ipu-miR-212
*tet3*	ccr-miR-199-5p/ola-miR-199a-5p
*tdg*	dre-miR-181a-2-3p, dre-miR-17a-2-3p

### BncRNA Is a Candidate for Dnmt Regulation

**FIGURE 7:**
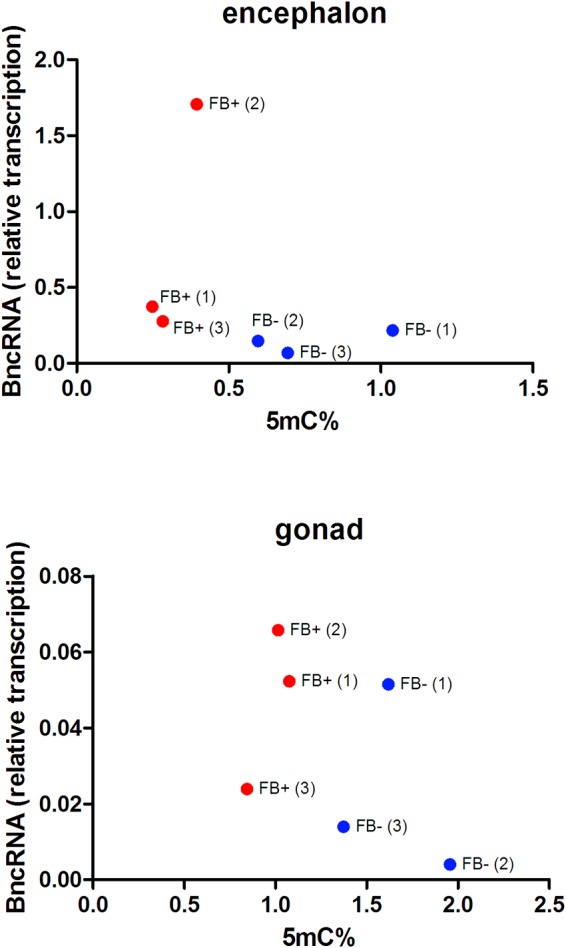
Comparison of the percentages of 5mC and transcriptional levels of BncRNA per sample in FB- females without B chromosome (blue, FB-) and females with B chromosome (red, FB+). Numbers inside parentheses indicate sample ID. Pearson’s correlation indicates inverse correlation between 5mC levels and BncRNA expression in encephalons (*R* = –0.3336 and *p* < 0.05) and ovaries (*R* = -0.4689 and *p* < 0.05) of FB+.

We performed RNA-protein interaction predictions based on software that considers only the sequence of the RNA and protein (RPIseq) and detected scores that indicated a positive interaction of both the complete BncRNA and region 2 of the BncRNA (Ramos et al., 2017) with Dnmt proteins (Supplementary Dataset 1). Similarly, we conducted predictions that considered sequence and structural information of RNA and protein (RPI-Pred) and again identified positive interactions. We also assessed the transcriptional status of BncRNA available in Ramos et al. (2017), which studied the same samples of the present study, and we correlated this data with the level of 5mC. This analysis revealed negative correlation in encephalon (*R* = -0.3336; *p* < 0.05) and ovaries (*R* = -0.4689; *p* < 0.05), suggesting that B chromosome promotes reduction of 5mC level by increase of BncRNA expression (Figure 7). Together, these data could indicate a positive interaction between the BncRNA and Dnmts.

## Discussion

### 5mC and 5hmC Localization on the B Chromosome

For decades, B chromosomes were thought of as non-functional units, since they are dispensable and heterochromatic (Camacho, 2005). However, evidence of transcriptionally active B-sequences has been emerging from several organisms (Miao et al., 1991; Carchilan et al., 2007; Trifonov et al., 2013; Li et al., 2017; Ma et al., 2017). Despite the discovery of these expressed sequences in B chromosomes, the inactivation of these elements should be necessary to avoid dosage effects, like those observed in aneuploidies (Han et al., 2007). Thus, DNA methylation could be a mechanism that acts in the silencing of specific genic regions or at the chromosomal level.

The B chromosome is completely enriched by 5mC marks in the canid *Nyctereutes procyonoides*, which is believed to be a possible mechanism of silencing (Bugno-Poniewierska et al., 2014). However, our chromosomal immunolocalization of 5mC in *A. latifasciata* shows marks scattered over all A and B chromosomes. Likewise, a scattered and uniform pattern of 5mC marks in A and B chromosomes was also observed in *Secale cereale* (Carchilan et al., 2007; Pereira et al., 2016). In these cases, high DNA methylation level likely is not involved in chromosome inactivation. In turn, our line scan showed reduced 5mC marks in centromeric regions of most chromosomes, including the B, which is in agreement with previous results that revealed hypomethylation of the active centromeres, even those located in B chromosomes (Koo et al., 2011). This active epigenetic status of the B chromosome centromere can represent an important feature for the maintenance and transmission of this element.

At the sequence level, in the leaf tissue of *S. cereale*, the E3900 subtelomeric sequence repeat of the B chromosome is methylated (Langdon et al., 2000), as is the inactive centromeric tandem repeat Bd49 in *Brachycome dichromosomatica* (Leach et al., 1995). Moreover, in *Zea mays* the B-specific satellite ZmB is hypomethylated in the active centromere, while its inactive version is hypermethylated (Koo et al., 2011). In *Eyprepocnemis plorans* (López-León et al., 1991) and *Rattus rattus* (Stitou et al., 2000), ribosomal RNA genes localized in the B chromosome are silenced by 5mC marks. Therefore, although DNA methylation seems not to act at the chromosomal level of *A. latifasciata*, this modification could be important to the inactivation at the sequence level.

Similar patterns of 5mC observed among A and B chromosomes of *A. latifasciata* could indicate that this epimark has the same role in the regulation of both types of chromosomes. Therefore, since A chromosomes are active, this could also indicate functional activity of B chromosomes. This hypothesis can be supported by evidence of active B gene copies in this species (Valente et al., 2014). However, although DNA methylation may not act in B chromosome inactivation, other mechanisms, such as histone modifications and late replication, can be acting, as suggested for the supernumerary chromosome of *B. dichromosomatica* (Houben et al., 1997). In mouse, for example, Rens et al. (2010) observed hypomethylation both of active and inactive X chromosomes in females, which is not common in eutherian mammals, and the authors attributed the inactivation of one X chromosome to histone modifications. Therefore, other possible mechanisms of B chromosome inactivation in *A. latifasciata* need better investigation.

Chromosomal distribution of 5hmC is still poorly explored, with studies focused only on mammals (Szulwach et al., 2011; Kubiura et al., 2012; Li et al., 2013; Yamaguchi et al., 2013; Bogomazova et al., 2014; Efimova et al., 2015, 2017), which reported localization of this mark mostly in active regions, such as R-bands (Kubiura et al., 2012; Efimova et al., 2015) and the reactivated X chromosome (Bogomazova et al., 2014). Here, we conducted the first localization of 5hmC in chromosome spreads of a non-mammalian species and the first analysis focused on supernumerary B chromosomes. Our immunostaining of 5hmC showed marks scattered over all A chromosomes and on the B chromosome, without accumulation of this mark in any region, which can indicate that 5hmC does not act at the chromosome level. However, studies at the sequence level need to be performed to advance our understanding of 5hmC control of B chromosomes. Moreover, similar patterns of 5hmC distribution between A and B chromosomes can be further evidence of supernumerary chromosome activity, similarly to that discussed for 5mC marks.

The chromosomal profiles of 5mC and 5hmC of the B chromosome indicate that this element can be functional and impact the global epigenetic DNA modification status of the cell, which was confirmed in the quantification of 5mC and 5hmC. Therefore, the exploration of these effects is relevant to elucidate the mechanisms of transmission and maintenance of the B chromosome and to understand the consequences of epigenetic modifications.

### Impact of B Chromosomes in the Global Levels of 5mC and 5hmC

**FIGURE 8:**
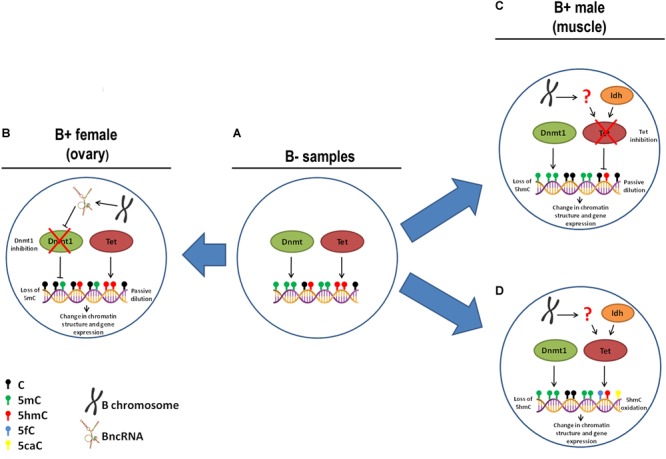
Hypothesis of causes of 5mC and 5hmC variations with B chromosome presence in *A. latifasciata*. **(A)** In individuals without B chromosomes, regular function of Dnmt and Tet is observed. **(B)** In ovary of FB+, the chromosome transcribes BncRNA, which can inhibit Dnmt1 and promote loss of 5mC due to passive dilution. **(C)** In muscle of MB+, the B chromosome reduces the inhibition of Tet function by an unidentified mechanism and promotes loss of 5mhC due to passive dilution. **(D)** Alternatively, in muscle of MB+, the B chromosome can regulate Tet by an unidentified mechanism, which promotes 5hmC oxidation.

To explore the molecular mechanisms underlying the loss of 5mC and 5hmC in some tissues of B+ individuals, we quantified the transcription levels of the genes related to epigenetic DNA modification (*dnmt1, dnmt3a, dnmt3b, tet1, tet2, tet3*, and *tdg*) and detected upregulation of *tet3* in muscle of FB+. However, we did not observe variations in the level of 5mC or 5hmC, indicating that these changes are not enough to impact epigenetic DNA modifications. Moreover, we explored the expression of candidate miRNAs targeting DNA modification genes and the transcription of the *idh1* and *idh2* genes (regulators of the DNA modification genes) in the presence of supernumerary chromosome, and no variation was observed. In ovaries of FB+, we did not observe 5mC reduction followed by 5hmC accumulation. Moreover, we did not observe substantial alterations in the transcription of *tet* genes, which can indicate that active modifications are not the mechanisms that promoted reduction of 5mC to 5hmC, so passive mechanisms might be responsible for this. Passive demethylation can be achieved by reduced activity of Dnmt1 during replication (Kohli and Zhang, 2013). However, we did not observe changes in the transcription of the *dnmt1* gene in these samples, indicating the possible occurrence of a posttranscriptional mechanism of regulation of Dnmt1, such as the action of miRNAs. In turn, we did not observe alteration in any candidate miRNA that would inhibit *dnmt1* RNA. In addition, another posttranscriptional mechanism of Dnmt1 inhibition can explain the interaction of Dnmt1 enzyme with RNAs. The association of the non-coding RNA ecCEBPA and Dnmt1 is involved in the reduction of Dnmt1 function in human (Di Ruscio et al., 2013). Interestingly, we observed positive interaction of BncRNA and Dnmt1 protein, which indicated that the BncRNA element, which is upregulated in B+ samples (Ramos et al., 2017), can act as an inhibitor of this enzyme, promoting passive DNA demethylation in ovaries of FB+ (Figure 8A,B). It is important to highlight that we found a tendency of reduction or increase in the level of 5mC and 5hmC and expression of some genes among the groups, but that was not statistically significant. This can be a consequence of the sample size.

With regard to loss of 5hmC in muscle of B+ males, this variation could be explained by reduction in the activity of Tet enzymes, but we did not observe differences in the transcription of *tet* genes or their candidate miRNA regulators. Moreover, we did not identify altered transcription of *idh1* and *idh2* genes, which encode cofactors of Tet enzymes. Therefore, two possible scenarios can explain the loss of 5hmC: passive dilution and 5hmC oxidation. In the first case, the B chromosome could express any factor that can inhibit Tet enzymes and avoid 5mC oxidation, while remaining 5hmC marks are lost during DNA replication (Figure 8A,C). In the second situation, the supernumerary chromosome could express any factor that stimulates Tet to convert 5hmC to 5fC and 5caC forms without 5mC conversion to 5hmC, which could explain the stable levels of 5mC between B- and B+ samples (Figure 8A,D).

### Sex Effects in DNA Modifications Routes

We observed reduced level of 5mC in ovaries compared to testes independent of B chromosome presence, similar to observed in *Danio rerio* (Laing et al., 2018). These differences can result of high proportion of maturing gametes in testes, which are known to be hypermethylated among vertebrates (Jiang et al., 2013; Potok et al., 2013; Laing et al., 2018). Although the reduced 5mC level in ovaries, we found opposite upregulation of *dnmt1* and *dnmt3b* in this organ. This can be partially explained by miRNA repression of *dnmt3b* since the miR-132b is upregulated in ovaries. Moreover, we observed evidences of increased level of 5hmC (although not statistically significant) and *tdg* transcription in ovaries, which can indicate active DNA demethylation, although increased transcription of *tet* genes has not been observed.

### Sex-Biased Effects of B Chromosomes

The epigenetic pattern of B chromosome presence observed in *A. latifasciata* was sex-associated, although B chromosomes occur in both sexes in this species. The B chromosome presence was correlated with global reduction of 5mC in ovaries of FB+ and reduction of 5hmC in muscle of MB+. Association of sex and B chromosomes has already been reported for *A. latifasciata*, as in the transcriptional variation of the non-coding BncRNA (Ramos et al., 2017) and the hnRNP Q-like gene (Carmello et al., 2017). Furthermore, the sex–B chromosome association has long been described in many species as diverse as fish and invertebrates (Camacho et al., 2011). Cichlid fish represent a promising model to investigate B chromosomes. The element has been described in 21 species of the group, and in eight species, all of the B-carrying individuals were female (Yoshida et al., 2011; Clark et al., 2017). Although the association of B chromosomes and sex is still enigmatic, we could hypothesize that in cichlids, the B chromosome bias for females could favor their drive during female meiosis. Considering that disturbances in the transmission of B chromosome during mitosis have been reported under DNA demethylation in rye (Neves et al., 1992), the sex variation detected for the *A. latifasciata* female B carriers could indicate that B chromosomes and sex physiology are in some way connected in this species.

## Conclusion

The B chromosome of *A. latifasciata* has a pattern of 5mC and 5hmC epimarks that can suggest its active status or that DNA methylation, at least, is not involved in B-silencing. Moreover, our data correlate B chromosome presence with passive DNA demethylation associated with sex, and the epigenetic effects of the B chromosome presence can also explain the previously reported variations in the transcription levels of several classes of RNA. B chromosomes represent additional chromatin in the nucleus, and their presence seems to have an extensive impact on several cellular processes, including epigenetic modification. The state of the art of B chromosome science suggests that besides B chromosomes favoring their own drive during cell division, these accessory elements seem to cause major impacts in the cell and in the organism.

## Ethics Statement

This study was carried out in accordance with the Brazilian College of Animal Experimentation and was approved by the Institute of Biosciences (UNESP) ethics committee on the use of animals (Protocol No. 486-2013 and 769-2015).

## Author Contributions

AC, BF, NV, BC, RO, and CM provided the substantial contributions to the conception of the work, wrote, read, and approved the manuscript. AC and NV performed the acquisition, analyses, and interpretation of cytogenetic data. AC and BC performed the nucleic acid acquisition and RNA expression analyses. AC and CM performed the epigenetic analyses and critically edited the final manuscript. AC and BF performed the bioinformatic analyses. AC and RO performed the statistical analyses.

## Conflict of Interest Statement

The authors declare that the research was conducted in the absence of any commercial or financial relationships that could be construed as a potential conflict of interest.

## Footnotes

1 http://sacibase.ibb.unesp.br/

2 http://cichlid.umd.edu/cichlidlabs/kocherlab/bouillabase.html

3 http://www.mirbase.org/

## References

[B1] Alvarez-Garcia I. Miska E. A. (2005). MicroRNA functions in animal development and human disease. *Development* 132 4653–4662. 10.1242/dev.02073 16224045

[B2] Anders S. Huber W. (2010). Differential expression analysis for sequence count data. *Genome Biol.* 11:R106. 10.1186/gb-2010-11-10-r106 20979621 PMC3218662

[B3] Arvinden V. R. Rao A. K. D. M. Rajkumar T. Mani S. (2017). Regulation and functional significance of 5-hydroxymethylcytosine in cancer. *Epigenomes* 1:19 10.3390/epigenomes1030019

[B4] Banaei-Moghaddam A. M. Martis M. M. Macas J. Gundlach H. Himmelbach A. Altschmied L. (2015). Genes on B chromosomes: old questions revisited with new tools. *Biochim. Biophys. Acta* 1849 64–70. 10.1016/j.bbagrm.2014.11.007 25481283

[B5] Bartel D. P. (2004). MicroRNAs: genomics, biogenesis, mechanisms, and function. *Cell* 116 281–297. 10.1016/S0092-8674(04)00045-5 14744438

[B6] Bertollo L. A. C. Takahashi C. S. Moreira-Filho O. (1978). Cytotaxonomic consideration on Hoplias lacerdae (Pisces, Erythrinidae). *Braz. J. Genet.* 1 103–120.

[B7] Beukeboom L. W. (1994). Bewildering Bs: an impression of the 1st B-Chromosome Conference. *Heredity* 73 328–336. 10.1038/hdy.1994.140

[B8] Bogomazova A. N. Lagarkova M. A. Panova A. V. Nekrasov E. D. Kiselev S. L. (2014). Reactivation of X chromosome upon reprogramming leads to changes in the replication pattern and 5hmC accumulation. *Chromosoma* 123 117–128. 10.1007/s00412-013-0433-x 23982752

[B9] Bugno-Poniewierska M. Solek P. Wronski M. Potocki L. Jezewska-Witkowska G. Wnuk M. (2014). Genome organization and DNA methylation patterns of B chromosomes in the red fox and Chinese raccoon dogs. *Hereditas* 151 169–176. 10.1111/hrd2.00049 25491428

[B10] Camacho J. P. M. (2005). “B chromosomes,” in *The Evolution of the Genome* ed. Gregory T. R. (San Diego, CA: Elsevier) 223–286. 10.1016/B978-012301463-4/50006-1

[B11] Camacho J. P. M. Schmid M. Cabrero J. (2011). B chromosomes and sex in animals. *Sex. Dev.* 5 155–166. 10.1159/000324930 21430369

[B12] Camacho J. P. M. Sharbel T. F. Beukeboom L. W. (2000). B-chromosome evolution. *Philos. T. R. Soc. B* 355 163–178. 10.1098/rstb.2000.0556 10724453 PMC1692730

[B13] Carchilan M. Delgado M. Ribeiro T. Costa-Nunes P. Caperta A. Morais-Cecílio L. (2007). Transcriptionally active heterochromatin in rye B chromosomes. *Plant Cell* 19 1738–1749. 10.1105/tpc.106.046946 17586652 PMC1955731

[B14] Carmello B. O. Coan R. L. B. Cardoso A. L. Ramos E. Fantinatti B. E. A. Marques D. (2017). The hnRNP Q-like gene is retroinserted into the B chromosomes of the cichlid fish *Astatotilapia latifasciata*. *Chromosome Res.* 25 277–290. 10.1007/s10577-017-9561-0 28776210

[B15] Cedar H. Bergman Y. (2009). Linking DNA methylation and histone modification: patterns and paradigms. *Nat. Rev. Genet.* 10 295–304. 10.1038/nrg2540 19308066

[B16] Clark F. E. Conte M. A. Ferreira-Bravo I. A. Poletto A. B. Martins C. Kocher T. (2017). Dynamic sequence evolution of a sex-associated B chromosome in Lake Malawi cichlid fish. *J. Hered.* 108 53–62. 10.1093/jhered/esw059 27630131

[B17] Coan R. L. B. Martins C. (2018). Landscape of transposable elements focusing on the B chromosome of the cichlid fish *Astatotilapia latifasciata*. *Genes* 9:269. 10.3390/genes9060269 29882892 PMC6027319

[B18] Costa G. Barra V. Lentini L. Cillufo D. Di Leonardo A. (2016). DNA demethylation caused by 5-aza-2′-deoxycytidine induces mitotic alterations and aneuploidy. *Oncotarget* 7 3726–3739. 10.18632/oncotarget.6897 26771138 PMC4826165

[B19] Denis H. Ndlovu M. N. Fuks F. (2011). Regulation of mammalian DNA methyltransferases: a route to new mechanisms. *EMBO Rep.* 12 647–656. 10.1038/embor.2011.110 21660058 PMC3128952

[B20] Di Ruscio A. Ebralidze A. K. Benoukraf T. Amabile G. Goff L. A. Terragni J. (2013). DNMT1-interacting RNAs block gene-specific DNA methylation. *Nature* 503 371–376. 10.1038/nature12598 24107992 PMC3870304

[B21] Efimova O. A. Pendina A. A. Tikhonov A. V. Fedorova I. D. Krapivin M. I. Chiryaeva O. G. (2015). Chromosome hydroxymethylation patterns in human zygotes and cleavage-stage embryos. *Reproduction* 149 223–233. 10.1530/REP-14-0343 25504867

[B22] Efimova O. A. Pendina A. A. Tikhonov A. V. Parfenyev S. E. Mekina I. D. Komarova E. M. (2017). Genome-wide 5-hydroxymethylcytosine patterns in human spermatogenesis are associated with semen quality. *Oncotarget* 8 88294–88307. 10.18632/oncotarget.18331 29179435 PMC5687605

[B23] Enright A. J. John B. Gaul U. Tuschl T. Sander C. Marks M. S. (2004). MicroRNAs targets in Drosophila. *Genome Biol.* 5:R1. 10.1186/gb-2003-5-1-r1 14709173 PMC395733

[B24] Fantinatti B. E. A. (2015). *Elucidação Dos Efeitos De Cromossomos Supernumeráveis Na Espécie De Ciclídeo Africano Astatotilapia latifasciata, Com Base Na Análise De Expressão De Micro-RNAs*. Doctoral Thesis (In Portuguese). Universidade Estadual Paulista, Instituto de Biociências de Botucatu.

[B25] Fantinatti B. E. A. Martins C. (2016). Development of chromosomal markers based on next-generation sequencing: the B chromosome of the cichlid fish *Astatotilapia latifasciata* as a model. *BMC Genet.* 17:119. 10.1186/s12863-016-0427-9 27539214 PMC4991083

[B26] Fantinatti B. E. A. Mazzuchelli J. Valente G. T. Cabral-de-Mello D. C. Martins C. (2011). Genomic content and new insights on the origin of the B chromosome of the cichlid fish Astatotilapia latifasciata. *Genetica* 139 1273–1282. 10.1007/s10709-012-9629-x 22286964

[B27] Figueroa M. E. Abdel-Wahab O. Lu C. Ward P. S. Patel J. Shih A. (2010). Leukemic IDH1 and IDH2 mutations result in a hypermethylation phenotype, disrupt TET2 function, and impair hematopoietic differentiation. *Cancer Cell* 18 553–567. 10.1016/j.ccr.2010.11.015 21130701 PMC4105845

[B28] Goll M. G. Bestor T. H. (2005). Eukaryotic cytosine methyltransferases. *Annu. Rev. Biochem.* 74 481–514. 10.1146/annurev.biochem.74.010904.153721 15952895

[B29] Goll M. G. Halpern M. E. (2011). DNA methylation in zebrafish. *Prog. Mol. Biol. Transl. Sci.* 101 193–218. 10.1016/B978-0-12-387685-0.00005-6 21507352 PMC5455991

[B30] Han F. Lamb J. C. Yu W. Gao Z. Birchler J. A. (2007). Centromere function and nondisjunction are independent components of the maize B chromosome accumulation mechanism. *Plant Cell* 19 524–533. 10.1105/tpc.106.049577 17322406 PMC1867328

[B31] Harrison A. Parle-McDermott A. (2011). DNA methylation: a timeline of methods and applications. *Front. Genet.* 2:74. 10.3389/fgene.2011.00074 22303369 PMC3268627

[B32] Head J. A. (2014). Patterns of DNA methylation in animals: an ecotoxicological perspective. *Integr. Comp. Biol.* 54 77–86. 10.1093/icb/icu025 24785828

[B33] Houben A. Belyaev N. D. Leach C. R. Timmis J. N. (1997). Differences of histone H4 acetylation and replication timing between A and B chromosomes of Brachycome dichromosomatica. *Chromosome Res.* 5 233–237. 10.1023/B:CHRO.0000032297.10876.86 9244450

[B34] Huang W. Du Y. Zhao X. Jin W. (2016). B chromosome contains active genes and impacts the transcription of A chromosomes in maize (*Zea mays* L.). *BMC Plant Biol.* 18:88. 10.1186/s12870-016-0775-7 27083560 PMC4833949

[B35] Ito S. Shen L. Dai Q. Wu S. C. Collins L. B. Swenberg J. A. (2011). Tet proteins can convert 5-methylcytosine to 5-formylcytosine and 5-carboxylcytosine. *Science* 333 1300–1303. 10.1126/science.1210597 21778364 PMC3495246

[B36] Jiang L. Zhang J. Wang J. J. Wang L. Zhang L. Li G. (2013). Sperm, but not oocyte, DNA methylome is inherited by zebrafish early embryos. *Cell* 153 773–784. 10.1016/j.cell.2013.04.041 23663777 PMC4081501

[B37] Jones N. (2017). New species with B chromosomes discovered since 1980. *Nucleus* 60 263–281. 10.1007/s13237-017-0215-6

[B38] Kertesz M. Iovino M. Unnerstall U. Gaul U. Segal E. (2007). The role of site accessibility in microRNA target recognition. *Nat. Genet.* 39 1278–1284. 10.1038/ng2135 17893677

[B39] Kohli R. M. Zhang Y. (2013). TET enzymes, TDG and the dynamics of DNA demethylation. *Nature* 502 472–479. 10.1038/nature12750 24153300 PMC4046508

[B40] Koo D. H. Han F. Birchler J. A. Jiang J. (2011). Distinct DNA methylation patterns associated with active and inactive centromeres of the maize B chromosome. *Genome Res.* 21 908–914. 10.1101/gr.116202.110 21518739 PMC3106323

[B41] Krüger J. Rehmsmeier M. (2006). RNAhybrid: microRNA target prediction easy, fast and flexible. *Nucleic Acids Res.* 34 W451–W454. 10.1093/nar/gkl243 16845047 PMC1538877

[B42] Kubiura M. Okano M. Kimura H. Kawamura F. Tada M. (2012). Chromosome-wide regulation of euchromatin-specific 5mC to 5hmC conversion in mouse ES cells and female human somatic cells. *Chromosome Res.* 20 837–848. 10.1007/s10577-012-9317-9 23111490 PMC3524505

[B43] Kuc C. Richard D. J. Johnson S. Bragg L. Servos M. R. Doxey A. C. (2017). Rainbow trout exposed to benzo[a]pyrene yields conserved microRNA binding sites in DNA methyltranferases across 500 milion years of evolution. *Sci. Rep.* 7:16843. 10.1038/s41598-017-17236-x 29203905 PMC5715007

[B44] Kurdyukov S. Bullock M. (2016). DNA methylation analyses: choosing the right method. *Biology* 5:3. 10.3390/biology5010003 26751487 PMC4810160

[B45] Laing L. V. Viana J. Dempster E. L. Uren-Webster T. M. van-Aerle R. Mill J. (2018). Sex-specific transcription and DNA methylation profiles of reproductive and epigenetic associated genes in the gonads and livers of breeding zebrafish. *Comp. Biochem. Physiol.* 222 16–25. 10.1016/j.cbpa.2018.04.004 29655816

[B46] Langdon T. Seago C. Jones R. N. Ougham H. Thomas H. Forster J. W. (2000). De novo evolution of satellite DNA on the rye B Chromosome. *Genetics* 154 869–884. 10655237 10.1093/genetics/154.2.869 PMC1460944

[B47] Leach C. R. Tamzin M. D. Franks T. K. Spiniello S. S. Hanrahan C. F. Timmis J. N. (1995). Organisation and origin of a B chromosome centromeric sequence from Brachycome dichromosomatica. *Chromosoma* 103 708–714. 10.1007/BF00344232 7664618

[B48] Li Y. Jing X. A. Aldrich J. C. Clifford C. Chen J. Akbari O. S. (2017). Unique sequence organization and small RNA expression of a “selfish” B chromosome. *Chromosoma* 126 753–768. 10.1007/s00412-017-0641-x 28780664

[B49] Li Y. Miyanari Y. Shirane K. Nitta H. Kunota T. Ohashi H. (2013). Sequence-specific microscopic visualization of DNA methylation status at satellite repeats in individual cell nuclei and chromosomes. *Nucleic Acids Res.* 41:e186. 10.1093/nar/gkt766 23990328 PMC3799461

[B50] López-León M. D. Cabrero J. Camacho J. P. M. (1991). A nucleolus organizer region in a B chromosome inactivated by DNA methylation. *Chromosoma* 100 134–138. 10.1007/BF00418247

[B51] Ma W. Gabriel T. S. Martis M. M. Gursinky T. Schubert V. Vrána J. (2017). Rye B chromosomes encode a functional Argonaute-like protein with in vitro slicer activities similar to its A chromosome paralog. *New Phytol.* 213 916–928. 10.1111/nph.14110 27468091

[B52] Martis M. M. Klemme S. Banaei-Moghaddam A. M. Blattner F. R. Macas J. Schmutzer T. (2012). Selfish supernumerary chromosome reveals its origin as a mosaic of host genome and organellar sequences. *Proc. Natl. Acad. Sci. U.S.A.* 109 13343–13346. 10.1073/pnas.1204237109 22847450 PMC3421217

[B53] Mei Q. Li X. Meng Y. Wu Z. Guo M. Zhao Y. (2012). A facile and specific assay for quantifying microRNA by an optimized RT-qPCR approach. *PLoS One* 7:e46890. 10.1371/journal.pone.0046890 23071657 PMC3465266

[B54] Mendioroz M. Do C. Jiang X. Liu C. Darbary H. K. Lang C. F. (2015). Trans effects of chromosome aneuploidies on DNA methylation patterns in human Down syndrome and mouse models. *Genome Biol.* 16:263. 10.1186/s13059-015-0827-6 26607552 PMC4659173

[B55] Miao V. P. Covert S. F. VanEtten H. D. (1991). A fungal gene for antibiotic resistance on a dispensable (”B”) chromosome. *Science* 254 1773–1776. 10.1126/science.1763326 1763326

[B56] Muller P. Y. Janovjak H. Miserez A. R. Dobbie Z. (2002). Processing of gene expression data generated by quantitative real-time RT-PCR. *Biotechniques* 32 1372–1374. 12074169

[B57] Muppirala U. K. Honavar V. G. Dobbs D. (2011). Predicting RNA-protein interactions using only sequence information. *BMC Bioinformatics* 12:489. 10.1371/journal.pone.0046890 22192482 PMC3322362

[B58] Navarro-Domínguez B. Martín-Peciña M. Ruiz-Ruano F. J. Cabrero J. Corral J. M. López-León M. D. (2019). Gene expression changes elicited by a parasitic B chromosome in the grasshopper *Eyprepocnemis plorans* are consistent with its phenotypic effects. *Chromosoma* 128 53–67. 10.1007/s00412-018-00689-y 30617552

[B59] Neves N. Barão A. Castilho A. Silva M. Morais L. Carvalho V. (1992). Influence of DNA methylation on rye B-chromosome non disjunction. *Genome* 35 650–652. 10.1139/g92-098

[B60] Pereira H. S. Delgado M. Viegas W. Rato J. M. Barão A. Caperta A. D. (2016). Rye (Secale cereale) supernumerary (B) chromosomes associated with heat tolerance during early stages of male sporogenesis. *Ann. Bot.* 119 325–337. 10.1093/aob/mcw206 27818381 PMC5314639

[B61] Plongthongkum N. Diep D. H. Zhang K. (2014). Advances in the profiling of DNA modifications: cytosine methylation and beyond. *Nat. Rev. Gen.* 15 647–661. 10.1038/nrg3772 25159599

[B62] Poletto A. B. Ferreia I. A. Martins C. (2010). The B chromosomes of the African cichlid fish Haplochromis obliquidens harbour 18S rRNA gene copies. *BMC Genet.* 11:1. 10.1186/1471-2156-11-1 20051104 PMC2806386

[B63] Potok M. E. Nix D. A. Parnell T. J. Cairns B. R. (2013). Reprogramming the maternal zebrafish genome after fertilization to match the paternal methylation pattern. *Cell* 153 759–772. 10.1016/j.cell.2013.04.030 23663776 PMC4030421

[B64] Ramos E. Cardoso A. L. Brown J. Marques D. F. Fantinatti B. E. A. Cabral-de-Mello D. C. (2017). The repetitive DNA element BncDNA, enriched in the B chromosome of the cichlid fish Astatotilapia latifasciata, transcribes a potentially noncoding RNA. *Chromosoma* 126 313–323. 10.1007/s00412-016-0601-x 27169573

[B65] Rens W. Wallduck M. S. Lovell F. L. Ferguson-Smith M. A. Ferguson-Smith A. (2010). Epigenetic modifications on X chromosomes in marsupial and monotreme mammals and implications for evolution of dosage compensation. *Proc. Natl. Acad. Sci. U.S.A.* 107 17657–17662. 10.1073/pnas.0910322107 20861449 PMC2955130

[B66] Ruban A. Schmutzer T. Scholz U. Houben A. (2017). How next-generation sequencing has aided our understanding of the sequence composition and origin of b chromosomes. *Genes* 8:294. 10.3390/genes8110294 29068386 PMC5704207

[B67] Sambrook J. Russel D. W. (2001). *Molecular Cloning. A Laboratory Manual, 3rd Edn*. Cold Spring Harbor, NY: Cold Spring Harbor Laboratory Press.

[B68] Shen L. Song C. X. He C. Zuang Y. (2014). Mechanism and function of oxidative reversal of DNA and RNA methylation. *Annu. Rev. Biochem.* 83 585–614. 10.1146/annurev-biochem-060713-035513 24905787 PMC4786441

[B69] Simon P. (2003). Q-Gene: processing quantitative real-time RT-PCR data. *Bioinformatics* 19 1439–1440. 10.1093/bioinformatics/btg157 12874059

[B70] Stitou S. Díaz-de-la-Guardia R. Jiménez R. Burgos M. (2000). Inactive ribosomal cistrons are spread throughout the B chromosomes of Rattus rattus (Rodentia, Muridae). Implications for their origin and evolution. *Chromosome Res.* 8 305–311. 10.1023/A:1009227427575 10919721

[B71] Suresh V. Liu L. Adjeroh D. Zhou X. (2015). RPI-Pred: predicting ncRNA-protein interaction using sequence and structural information. *Nucleic Acids Res.* 43 1370–1379. 10.1093/nar/gkv020 25609700 PMC4330382

[B72] Szulwach K. E. Li X. Li Y. Song C. X. Han J. W. Kim S. S. (2011). Integrating 5-hydroxymethylcytosine into the epigenomic landscape of human embryonic stem cells. *PLoS Genet.* 7:e1002154. 10.1371/journal.pgen.1002154 21731508 PMC3121778

[B73] Trifonov V. A. Dementyeva P. V. Larkinm D. M. O’Brien P. C. M. Perelman P. L. Yang F. (2013). Transcription of a protein-coding gene on B chromosomes of the Siberian roe deer (Capreolus pygargus). *BMC Biology* 11:90. 10.1186/1741-7007-11-90 23915065 PMC3751663

[B74] Turcan S. Rohle D. Goenka A. Walsh L. A. Fang F. Yilmaz E. (2012). IDH1 mutation is sufficient to establish the glioma hypermethylator phenotype. *Nature* 483 479–483. 10.1038/nature10866 22343889 PMC3351699

[B75] Valente G. Nakajima R. Fantinatti B. E. A. Marques D. F. Almeida R. O. Simões R. P. (2017). B chromosomes: from cytogenetics to system biology. *Chromosoma* 126 73–81. 10.1007/s00412-016-0613-6 27558128

[B76] Valente G. T. Conte M. A. Fantinatti B. E. A. Cabral-de-Mello D. C. Carvalho R. F. Vicari M. R. (2014). Origin and evolution of B chromosomes in the cichlid fish Astatotilapia latifasciata based on integrated genomic analyses. *Mol. Biol. Evol.* 31 2061–2072. 10.1093/molbev/msu148 24770715

[B77] Varriale A. Bernardi G. (2006). DNA methylation and body temperature in fishes. *Gene* 385 111–121. 10.1016/j.gene.2006.05.031 17067764

[B78] Yamaguchi S. Hong K. Liu R. Inoue A. Shen L. Zhang K. (2013). Dynamics of 5-methylcytosine and 5-hydroxymethylcytosine during germ cell reprogramming. *Cell Res.* 23 329–339. 10.1038/cr.2013.22 23399596 PMC3587712

[B79] Yoshida K. Terai Y. Mizoiri S. Aibara M. Nishihara H. Watanabe M. (2011). B chromosomes have a functional effect on female sex determination in Lake Victoria cichlid fishes. *PLoS Genet.* 7:e1002203. 10.1371/journal.pgen.1002203 21876673 PMC3158035

