# Supplementary material for: Epigenetic DNA Modifications Are Correlated With B Chromosomes and Sex in the Cichlid Astatotilapia latifasciata
Source: Front Genet. 2019 Apr 12;10:324. doi: 10.3389/fgene.2019.00324 (PMC6474290; doi:10.3389/fgene.2019.00324)

***Supplementary Material***

**1 SUPPLEMENTARY TABLES AND FIGURES**

**1.1 Tables**

**Table S1.** Primers used to assess the transcription of DNA modification genes and miRNAs.

| **Primer ID** | **Primer sequence (5’-3’)** |
| --- | --- |
| ubce-f | GTCCGTTTCAATCCCAACTT |
| ubce-r | GTTCTCCGTCATCAGAGACT |
| dnmt1-f | CACCTCCAGCAGTTCATCGT |
| dnmt1-r | AGCAGACCCAGTGCAAACTT |
| dnmt3a-f | TGTCGCCATCGAAGGAGAAG |
| dnmt3a-r | AACCAGCTCGCCGATGCCAA |
| dnmt3b-f | GTTCAGGTGCGTGCCACTGT |
| dnmt3b-r | GATACCATTTGTGCTGAAGGC |
| tet1-f | GGAGTCTGTGTATGAGGTAT |
| tet1-r | GATAAGGTGGTTCCAGAAAGTG |
| tet2-f | GAATGCTCTGGTGGTGTTGC |
| tet2-r | AGAGCAATCCAATGGCAGGA |
| tet3-f | ACCACTGTTCACAAAGTTAC |
| tet3-r | TCATTAGGGTACTGATTCAC |
| tdg-f | GTGCTCCTTCTGTATTTTCA |
| tdg-r | GATGCCAAGAGGCTGGCAAT |
| idh1-f | CCACCTGTGCCCTCAAACTC |
| idh1-r | GATCTATGAGAAAGAGTATCGTGCTC |
| idh2-f | CCGAGAAGCTCATCCTGACC |
| idh2-r | GCTAAACTCTTCAACTCTGGCTTC |
| SL-poly(A) | GTCGTATCCAGTGCAGGGTCCGAGGTATTCGCACTGGATACGACAAAAAAAAAAAAAAAAAAVN |
| Universal-r | GCAGGGTCCGAGGTATTCG |
| U6-f | ATGACACGCAAATTCGTGAAG |
| miR-17a-2-3p-f | CCAGTGGAGGCACTTCAAGCTTT |
| miR-29b-f | CCTAGCACCATTTGAAATCAGTGTTT |
| miR-30b-2-3p-f | CTTTTCAGTCGGATGTTTGCAGC |
| miR-132b-f | AGACCATGGCTGTAGACTGTTACC |
| miR-181a-2-3p-f | CCATCGACCGTTGACTGTACCT |
| miR-199a-5p-f | GCCCAGTGTTCAGACTACCTGTTT |

**Table S2**. Expression level average (DESeq) of the predicted candidate miRNAs on the regulation of the DNA modification genes in small RNA-seq libraries. The miRNA with expression validated by RT-qPCR are highlighted in yellow. Base mean – mean normalised counts; Base mean (B-) – mean normalised counts from B- samples; Base mean (B+) – mean normalised counts from B+ samples; Fold Change – fold change from condition B- to B+; log2 Fold Change – the logarithm (to basis 2) of the fold change; p value – p value for the statistical significance of this change.

|  | ID | Base Mean | Base Mean (B-) | Base Mean (B+) | Fold Change | log2 Fold Change | p value |
| --- | --- | --- | --- | --- | --- | --- | --- |
| Encephalon (Male) | ssa-let-7b-3p | 0.3975 | 0.7949 | 0 | 0 | -Inf | 0.7463 |
|  | dre-miR-17a-2-3p | 0.1183 | 0.2365 | 0 | 0 | -Inf | 0.9876 |
|  | dre-miR-29b | 532.1357 | 508.4652 | 555.8063 | 1.0931 | 0.1284 | 0.9172 |
|  | ssa-miR-30d-2-3p | 79.5363 | 94.6623 | 64.4103 | 0.6804 | -0.5555 | 0.6678 |
|  | dre-miR-34b | 0.1198 | 0.2395 | 0 | 0 | -Inf | 0.9948 |
|  | ccr-miR-132b | 46.9827 | 42.2017 | 51.7637 | 1.2266 | 0.2946 | 0.7170 |
|  | ssa-miR-143-5p | 61.0017 | 62.0224 | 59.9810 | 0.9671 | -0.0483 | 0.9090 |
|  | dre-miR-181a-2-3p | 3411.3875 | 3641.8132 | 3180.9618 | 0.8735 | -0.1952 | 0.8488 |
|  | dre-miR-181b-5p | 10535.2042 | 11912.9762 | 9157.4322 | 0.7687 | -0.3795 | 0.7040 |
|  | ccr-miR-199-5p | 12547.0538 | 11012.8361 | 14081.2714 | 1.2786 | 0.3546 | 0.5824 |
|  | ola-miR-199a-5p | 4027.7290 | 2973.4258 | 5082.0322 | 1.7092 | 0.7733 | 0.2844 |
|  | ipu-miR-212 | 35002.1201 | 40166.0375 | 29838.2026 | 0.7429 | -0.4288 | 0.5826 |
| Encephalon (Female) | ssa-let-7b-3p | 0.1097 | 0.2193 | 0 | 0 | -Inf | 1 |
|  | dre-miR-17a-2-3p | 0.1106 | 0.2212 | 0 | 0 | -Inf | 1 |
|  | dre-miR-29b | 541.3492 | 550.0186 | 532.6798 | 0.9685 | -0.0462 | 0.9532 |
|  | ssa-miR-30d-2-3p | 60.9804 | 61.0008 | 60.9599 | 0.9993 | -0.0010 | 0.9875 |
|  | dre-miR-34b | 0.2421 | 0.0000 | 0.4842 | Inf | Inf | 0.9205 |
|  | ccr-miR-132b | 37.7213 | 35.6312 | 39.8115 | 1.1173 | 0.1600 | 0.8533 |
|  | ssa-miR-143-5p | 64.4791 | 68.5546 | 60.4037 | 0.8811 | -0.1826 | 0.8290 |
|  | dre-miR-181a-2-3p | 2612.3886 | 2383.2388 | 2841.5384 | 1.1923 | 0.2537 | 0.7717 |
|  | dre-miR-181b-5p | 7657.8540 | 6210.7831 | 9104.9248 | 1.4660 | 0.5519 | 0.4700 |
|  | ccr-miR-199-5p | 15362.2570 | 8469.8799 | 22254.6340 | 2.6275 | 1.3937 | 0.0401 |
|  | ola-miR-199a-5p | 5395.4049 | 2975.1345 | 7815.6753 | 2.6270 | 1.3934 | 0.0392 |
|  | ipu-miR-212 | 25907.5606 | 24326.8018 | 27488.3194 | 1.1300 | 0.1763 | 0.8237 |
| Muscle (Male) | ssa-let-7b-3p | 0.2359 | 0 | 0.4717 | Inf | Inf | 1 |
|  | dre-miR-17a-2-3p | 0 | 0 | 0 | NA | NA | NA |
|  | dre-miR-29b | 228.1925 | 232.0954 | 224.2896 | 0.9664 | -0.0494 | 0.9605 |
|  | ssa-miR-30d-2-3p | 111.4882 | 111.4528 | 111.5237 | 1.0006 | 0.0009 | 0.9513 |
|  | dre-miR-34b | 0 | 0 | 0 | NA | NA | NA |
|  | ccr-miR-132b | 3.0562 | 2.9073 | 3.2050 | 1.1024 | 0.1406 | 1 |
|  | ssa-miR-143-5p | 174.7197 | 185.5261 | 163.9134 | 0.8835 | -0.1787 | 0.9173 |
|  | dre-miR-181a-2-3p | 1396.6915 | 1385.3383 | 1408.0447 | 1.0164 | 0.0235 | 1 |
|  | dre-miR-181b-5p | 1683.8882 | 1647.6028 | 1720.1735 | 1.0440 | 0.0622 | 0.8843 |
|  | ccr-miR-199-5p | 419512.7892 | 342588.8741 | 496436.7043 | 1.4491 | 0.5351 | 0.6624 |
|  | ola-miR-199a-5p | 170861.7532 | 147806.2942 | 193917.2121 | 1.3120 | 0.3917 | 0.8203 |
|  | ipu-miR-212 | 119.0315 | 117.3603 | 120.7026 | 1.0285 | 0.0405 | 0.7175 |
| Muscle (Female) | ssa-let-7b-3p | 0 | 0 | 0 | NA | NA | NA |
|  | dre-miR-17a-2-3p | 0.2837 | 0 | 0.5673 | Inf | Inf | 0.9955 |
|  | dre-miR-29b | 216.4420 | 156.6882 | 276.1958 | 1.7627 | 0.8178 | 0.4057 |
|  | ssa-miR-30d-2-3p | 99.3772 | 129.6888 | 69.0655 | 0.5325 | -0.9090 | 0.6107 |
|  | dre-miR-34b | 0 | 0 | 0 | NA | NA | NA |
|  | ccr-miR-132b | 3.4462 | 5.3851 | 1.5074 | 0.2799 | -1.8369 | 0.3279 |
|  | ssa-miR-143-5p | 100.4028 | 86.2740 | 114.5316 | 1.3275 | 0.4087 | 0.7469 |
|  | dre-miR-181a-2-3p | 1374.0056 | 1546.9662 | 1201.0450 | 0.7764 | -0.3652 | 0.6875 |
|  | dre-miR-181b-5p | 1754.4715 | 1648.4308 | 1860.5123 | 1.1287 | 0.1746 | 0.8287 |
|  | ccr-miR-199-5p | 594260.1469 | 764069.6618 | 424450.6320 | 0.5555 | -0.8481 | 0.3687 |
|  | ola-miR-199a-5p | 167547.9645 | 177850.9356 | 157244.9935 | 0.8841 | -0.1777 | 0.8611 |
|  | ipu-miR-212 | 73.0057 | 84.5320 | 61.4794 | 0.7273 | -0.4594 | 0.7566 |
| Gonad (Male) | ssa-let-7b-3p | 0.3347 | 0 | 0.6694 | Inf | Inf | 1 |
|  | dre-miR-17a-2-3p | 0.5518 | 0 | 1.1036 | Inf | Inf | 1 |
|  | dre-miR-29b | 266.8375 | 232.5284 | 301.1466 | 1.2951 | 0.3731 | 0.6864 |
|  | ssa-miR-30d-2-3p | 175.3930 | 165.4205 | 185.3655 | 1.1206 | 0.1642 | 0.8632 |
|  | dre-miR-34b | 0 | 0 | 0 | NA | NA | NA |
|  | ccr-miR-132b | 0.4813 | 0.9626 | 0 | 0 | -Inf | 0.9171 |
|  | ssa-miR-143-5p | 1598.8876 | 1735.2418 | 1462.5334 | 0.8428 | -0.2466 | 0.7622 |
|  | dre-miR-181a-2-3p | 2282.9920 | 2462.5056 | 2103.4785 | 0.8542 | -0.2273 | 0.8103 |
|  | dre-miR-181b-5p | 3055.8382 | 3748.8926 | 2362.7839 | 0.6302 | -0.6659 | 0.4060 |
|  | ccr-miR-199-5p | 55811.2947 | 58521.0320 | 53101.5575 | 0.9073 | -0.1402 | 0.7786 |
|  | ola-miR-199a-5p | 16060.7066 | 16691.4613 | 15429.9520 | 0.9244 | -0.1133 | 0.8082 |
|  | ipu-miR-212 | 35.9800 | 51.3018 | 20.6582 | 0.4026 | -1.3122 | 0.1237 |
| Gonad (Female) | ssa-let-7b-3p | 0.3481 | 0 | 0.6962 | Inf | Inf | 0.8503 |
|  | dre-miR-17a-2-3p | 0.2863 | 0.5727 | 0 | 0 | -Inf | 1 |
|  | dre-miR-29b | 271.4620 | 235.1842 | 307.7398 | 1.3085 | 0.3879 | 0.4079 |
|  | ssa-miR-30d-2-3p | 274.2797 | 186.2899 | 362.2694 | 1.9447 | 0.9595 | 0.7582 |
|  | dre-miR-34b | 0 | 0 | 0 | NA | NA | NA |
|  | ccr-miR-132b | 0.3123 | 0 | 0.6246 | Inf | Inf | 1 |
|  | ssa-miR-143-5p | 1309.3738 | 1460.2929 | 1158.4548 | 0.7933 | -0.3341 | 0.9539 |
|  | dre-miR-181a-2-3p | 2512.2136 | 1517.7552 | 3506.6720 | 2.3104 | 1.2082 | 0.3538 |
|  | dre-miR-181b-5p | 4106.1883 | 2700.8485 | 5511.5281 | 2.0407 | 1.0290 | 0.4956 |
|  | ccr-miR-199-5p | 49584.4660 | 57103.0682 | 42065.8638 | 0.7367 | -0.4409 | 0.8801 |
|  | ola-miR-199a-5p | 12417.0259 | 12538.7249 | 12295.3269 | 0.9806 | -0.0283 | 0.5864 |
|  | ipu-miR-212 | 31.3496 | 41.9197 | 20.7796 | 0.4957 | -1.0125 | 0.1387 |

**1.2 Figures**


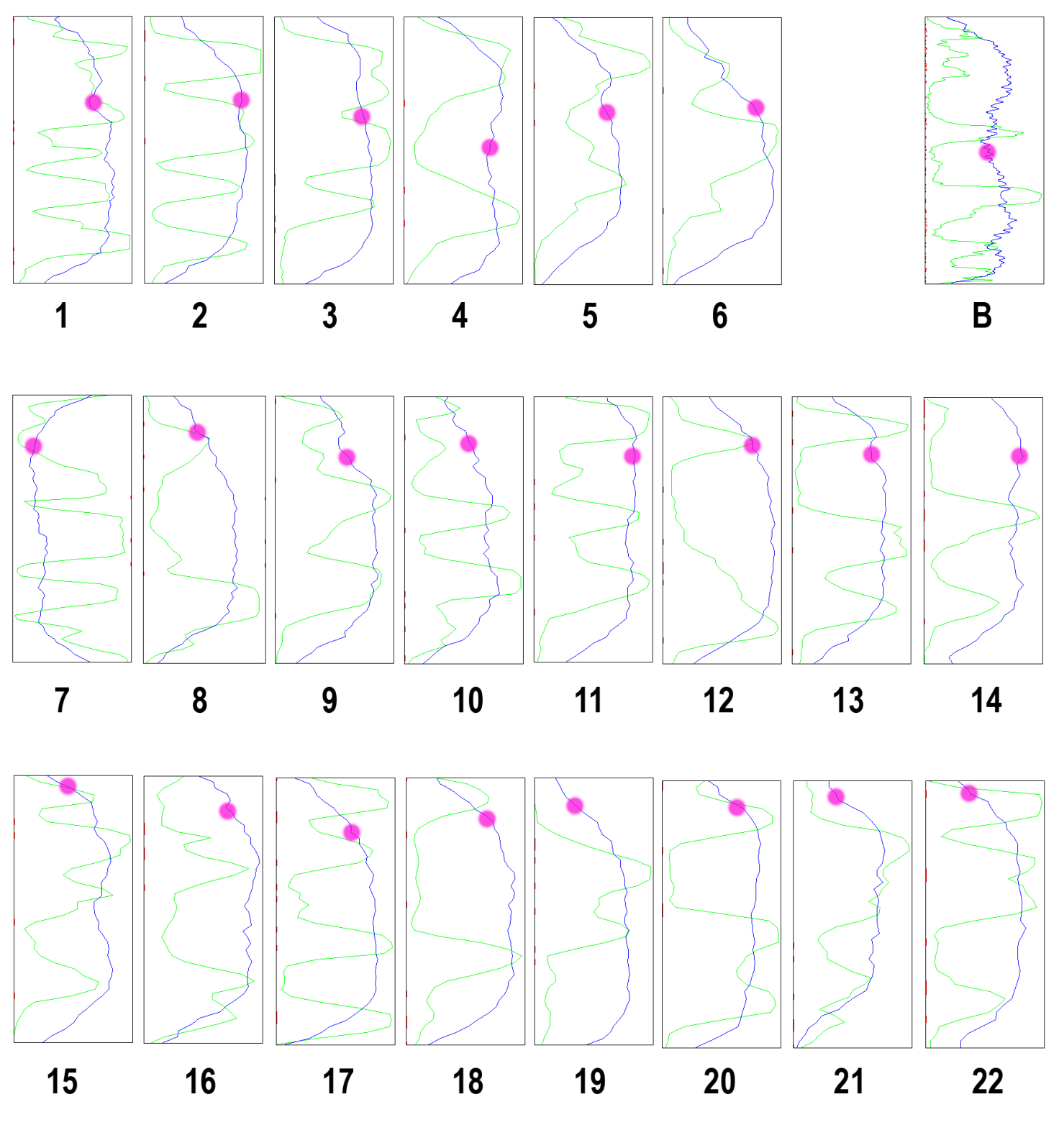


**Figure S1.** Methylation status of *Astatotilapia latifasciata* chromosomes of a representative cell. X-axis indicates fluorescence intensity and Y-axis represents chromosome size. Green lines indicate the distribution of 5mC along the chromosomes and blue lines represent the DAPI staining. Purple dots indicate the centromere. The numbers correspond to chromosome pairs and the B to B chromosome.

**Figure S2.** Hydroxymethylation status of *Astatotilapia latifasciata* chromosomes of a representative cell. X-axis indicates fluorescence intensity and Y-axis represents chromosome size. Red lines indicate the distribution of 5hmC along the chromosome and blue lines represent the DAPI staining. Purple dots indicate the centromere. The numbers correspond to chromosome pairs and the B to B chromosome.
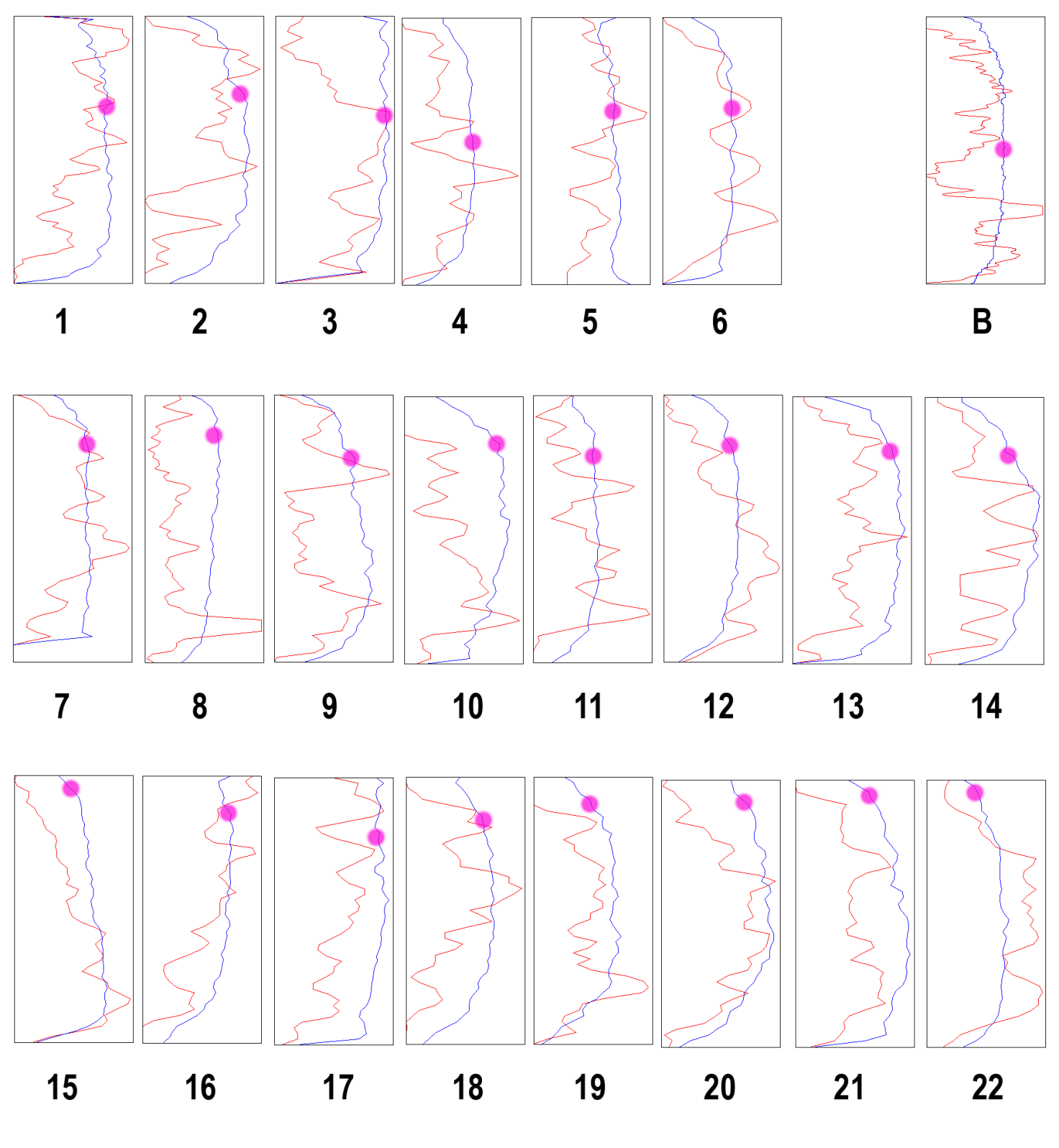


**2 SUPPLEMENTARY DATA**

**2.1 Supplementary dataset 1.** Protein-RNA interaction prediction using the software RPISeq and RPI-Pred. a) input sequences: complete sequence and subregion 2 of BncRNA and Dnmt1, Dnmt3a and Dnmt3b proteins sequence; b) Results of the RPISeq and RPI-Pred obtained from the interaction of the complete region and subregion 2 of BncRNA and Dnmt1.

*Prediction of RNA-protein interaction*

1. Input Sequences:

>BncRNA_complete_sequence

AUCAAUGUGUCCAGAACAUGCGGAAGUACUCACGUGACUGACUUUGUUUACAACUUAAGAACUGUAAGUUCAAUGCAUUCUAAUUUAUUUUCGGAGCCUUAUCCCGAACGAUUAUCGCCAGAAAACAGGUCAGUAAAACUGAAGGCCAGUGCAGAUACUACCACGAAAGCUAAAAGUGUUAAGAUAAAGUUGAAUUUUGAUGUAGAAGGCUCAGAGGAAAGUGAGAAACUAAUGUAUGCAGGAUUACCCGCUCCAGCACAGCAAGGAAGUUUCAGAGGAUGGAAGGAUAAAAGUAGAGAUAUGUGUUUUUAUUGUGGCUCAGUUUUCCAGCAGCCGCAGACACCCAGAGCAAAAAUGAAUUUGUGUAUUUCAUUUAACACAAAGGGUGAAAUGUUUGCUGACACUGAUAACAGCUUAUGUUUUCUCUGCCUUAUGUACCAUGGAGAAGGAACAUCCUCUGUGUCAGCUGGACCCUCAGCAGAGAAUGAACUCAACAUCCUUUUUGUCCGGUACUCUAUGUCACACACUGUGCCAAAAAACGUGUACAGACGCCACUACCAGGUAAUCCCAUGACUGGGAGCCACACUUUUGUUUCUCUCACACUGUUCAACAACAUGAUAACUGCUCACACAAGUGAAGGUCAAACAGUGAUUUUGUCACAUAACUCAGACAUAGUUUAUGACAUAACUAUACCAUGUGGACACAUUGAUCAUCG

>BncRNA_subregion_2

UGAUGUAGAAGGCUCAGAGGAAAGUGAGAAACUAAUGUAUGCAGGAUUACCCGCUCCAGCACAGCAAGGAAGUUUCAGAGGAUGGAAGGAUAAAAGUAG

>dnmt1_protein

MPTRTSLPLPDDVRKSLQRLDEEGSADEEHVKENLKLVQDFLHVDAQDQLTSLEEKMKSSEISKEVYISKVKAVLGKELHLENGSHSDDAEKNGKTNGFSNGSHKDEHDEDVTMSVQEEEESVKSPTSSKGKGGRRSKANSDTKKSPASTRVTRNSGKQPTLLSMFTKVQKRKSEDLNGEAVNGQNEPKKDDDVEESREEKRLKVESDDNAAPEESKSDIVKPVSAVKTPPPKCQDCRQYLDDSDLKFFQGDPDNALDEPEMLTNERLSLFDSNEDGFESYEDLPQHKITNFSVYDKRGHLCPFDSGLIEKNVELYFSCVVKPIYDDNPCMDGGVPAKKLGPINAWWITGFDGGEKALIGFTTAFADYILMQPSEEYAPIFALMQEKIYMSKIVVEFLQKNPDATYEDLLNKIETTVPPAGLNFNCFTEDTLLRHAQFVVEQVESYDEAGDSDEQPIIVTPCMRDLIKLAGVTLGKRRAARRQAIRHPTKIEKDSKGPTKATTTKLVYQIFDAFFSDQIEQNDKESAMKRQRCGVCEVCQSPDCGKCAACKDMIKFGGSGKSKQACRQRRCPNLAVKEAEDDENIEEEDVPVEKPKKVPHAKRKKQTQCKLTWIGESIHTEGKKQYYRKVSLNDELLEVGDCVSVSSEDPSIPLYLARITSMWEDNNGKMFHAHWFLRGIDTVLGETSDPLELVIVDECEDMLLNYVQGKVDVMYKAPSNNWFMEGGVDVDLKVIEDDGKSFFYQFWYDTEYARFEMPPKTSPSEECKFKFCGSCVRTKEREDKDKPRVFEPLENENHDTKALYAMACFKGEQFRVGDSVYLPPEAFNFSVKPASPVKRSHRKDDVDEDLYPEYYRKSSDYIKGSNLDAPEPFRVGRIKEIFCHRRSNGKPEMSDVKLRLYKFYRPENTHKGVKASYHTDINQLYWSDEEVTVNMGDVLGRCQVEYAEDLNESIQDYSSAGPERFYFLEAYNAKAKSFEDPPNHARSTVHKGKGKGKGKGKGKGKASAAQEQPDSQPKPKVPKYRTLDVFSGCGGLSEGFHQAAISETLWAIEMWEPAAQAFRLNNPGTTVFTEDCNILLKLVMSGEKTNSLGQKLPQKGDVEMLCGGPPCQGFSGMNRFNSRTYSKFKNSLVVSYLSYCDYYRPKFFLLENVRNFVSFKNSMVLKLTLRCLVRMGYQCTFGVLQAGQYGVAQTRRRAIILAAAPGEKLPRYPEPLHVFAPRACSLSVVVGEKRYVSNVTRGNGGIYRTITVRDTMSDLPEIRNGAAALEISYNGEPQSWFQRQIRGTQYQPILRDHICKDMSALVEGRMRYIPLAPGSDWRDLPNIEVRLKDGTLTKKLRYTHHDKKNGRSGTGALRGVCTCAGGKPCDPADRQFNTLIPWCLPHTGNRHNHWAGLYGRLEWDGFFSTTVTNPEPMGKQGRVLHPEQHRVVSVRECARSQGFPDTYRFFGNILDKHRQVGNAVPPPLSRAIGLEIKRCITERMKEEQASENIKQEKMELSD

>dnmt3a_protein

MMPSNTVTNTTTTADSPGNPMGERTILDSDLMEEKSPKSSKPGRKRKQFSVESRGSLKDSASVGHSYTMAMAQVFNGDMGGIRDRMPDACFPKQEKREVENGIPRDPSSCRAEDSSQGQSLSHPQENGFLSSREEQDLEKANDDCLMTPRKKRGRRKLERPTKYVEHKEEDGSDALKTEGGRGRLRGGVGWEISLRQRPMPRITFQAGDPYYISKRTREELLAKWKMEAEKKAKQMSAMNAMKDHEDNETETRNEEVSIIKHPQPSKQQQQPQQQQLQPQPHTKSQSQPQYQQQSPLVLPQQQQQLQHQQPPQQQQQQQQPTDPASPTVATTPEPVAIEGEDKTSPKSPDTESEYEDGRGFGIGELVWGKLRGFSWWPGRIVSWWMTGRSRAAEGTRWVMWFGDGKFSVVCVEKLLPLSSFNNAFHQPTYNKQPMYRKAIYEVLQVASSRAGKAFMACPDSDETETSKSVEMLNKQMIEWAMTGFQPTGPKGLEPPEEERNPYKEVYPEIWVEPEAAAYTPPPAKKPRKSTAEKPKVKDIIDERTRERLVYEVRQKCRSLEDICISCGSLNVSLEHPLFAGGMCQSCKNCFLECAYQYDDDGYQSYCTICCGGREVLMCGNNNCCRCFCVECVDLLVGQGAAHAAIKEDPWNCYMCGQKSVFGLLERRSDWPSRLQRFFANNHDQDFDPPKLYPPVMAEKRKPIRVLSLFDGIATGLLVLKELGIQVGRYVASEVCEDSITVGIVRHEGRIMYVGDVRNITRKHINEWGPFDLVIGGSPCNDLSIVNPARKGLYEGTGRLFFEFYRLLHEARPKQGEDRPFFWLFENVVAMGVSDKRDISRFLECNPVMIDAKEVSAAHRARYFWGNLPGMNRPLTAMCTDRLELQDCLEHGRTAKFGKVRTITTRSNSIKQGKDQHFPVYMNEKEDILWCTEMERVFGFPVHYTDVSNMSRLARQRLLGRSWSVPVIRHLFAPLKDYFACD

>dnmt3b_protein

MVMFEKESAQTRDQSSATAMPSNKYSAAIMEESNNMTATAAVNGDTPPAEGLSENDSGVELTNENSPLTAAEPPSPFSPKQNGDAASPQDGNQCSRGSRKRSRKRREDEESTWDSDKSSGASQLGLRQTPRPRTIFQAGLTPHTHGKPRRQNRKQEHGTSPCAGNPRAAAVGSGGVPETPRLELMEQDSKDSAQSTSTSSSSETKQEYSDNKGFGIGELVWGKIKGFSWWPGIVVTWRATGKRQASHGMRWLQWFGDGKFSEVSADKLDSLTAFPKFFSQASYTKLASYRRAVFQALEMASIRAEKTFPPCKSSNPEDQVKPMLDWANGGFLPKGGEGLKPTHSANSNPLDHHVLDVSLSEYFPSTKRPKLSLCKSKAAPEEMCNREQMVNEVLKNKRSIEEFCLSCGKTSATFHPLFEGGLCLTCKDVYLEMSYMYDDDGYQSYCTVCCGGREVLLCGNVNCCRCFCVDCLDILVDPGASDQARYLDPWRCYMCQPLLQYGVLKRRHDWSLKLQEFFANDNGQEFEKPKIYPAVPAEQRRPIRVLSLFDGIATGYLVLRDLGFKVGQYVASEVCEDSISVGVVRHEGKIKYVHDVRNITKKNIQEWGPFDLVIGGSPCNDLSIVNPARKGLYEGTGRLFFEFYRLLSEAKPKEGENRPFFWMFENVVAMAVNDKRDISRFLECNPVMIDAIEVSAAHRARYFWGNLPGMNRPLCASGMDKLQLQDCLDHGRVAKFGKVRTITTRSNSIKQGKDQHFPVLMNGKEDILWCTELERIFGFPVHYTDVSNMGRGARQKLLGRSWSVPVIRHLFAPLKDYFACE

1. Results:

*RPISeq software*

DNMT1

Interaction probabilities **BncRNA_complete_sequence + dnmt1_protein**

Prediction using RF classifier **0.7**

Prediction using SVM classifier **0.98**

What do these probabilities mean?

Interaction probabilities generated by RPISeq range from 0 to 1. In performance evaluation experiments, predictions with probabilities > 0.5 were considered “positive,” i.e., indicating that the corresponding RNA and protein are likely to interact. Using this threshold, accuracies of the classifiers ranged from 87 - 90% in cross-validation evaluation experiments on benchmark datasets. When classifiers were tested on independent (blind) datasets of RPIs, accuracies of the classifiers ranged from 57 – 99%.

Interaction probabilities **BncRNA_subregion2 + dnmt1_protein**

Prediction using RF classifier **0.55**

Prediction using SVM classifier **0.94**

What do these probabilities mean?

Interaction probabilities generated by RPISeq range from 0 to 1. In performance evaluation experiments, predictions with probabilities > 0.5 were considered “positive,” i.e., indicating that the corresponding RNA and protein are likely to interact. Using this threshold, accuracies of the classifiers ranged from 87 - 90% in cross-validation evaluation experiments on benchmark datasets. When classifiers were tested on independent (blind) datasets of RPIs, accuracies of the classifiers ranged from 57 – 99%.

DNMT3A

Interaction probabilities **BncRNA_complete_sequence + dnmt3a_protein**

Prediction using RF classifier **0.7**

Prediction using SVM classifier **0.98**

What do these probabilities mean?

Interaction probabilities generated by RPISeq range from 0 to 1. In performance evaluation experiments, predictions with probabilities > 0.5 were considered “positive,” i.e., indicating that the corresponding RNA and protein are likely to interact. Using this threshold, accuracies of the classifiers ranged from 87 - 90% in cross-validation evaluation experiments on benchmark datasets. When classifiers were tested on independent (blind) datasets of RPIs, accuracies of the classifiers ranged from 57 – 99%.

Interaction probabilities **BncRNA_subregion2 + dnmt3a_protein**

Prediction using RF classifier **0.7**

Prediction using SVM classifier **0.93**

What do these probabilities mean?

Interaction probabilities generated by RPISeq range from 0 to 1. In performance evaluation experiments, predictions with probabilities > 0.5 were considered “positive,” i.e., indicating that the corresponding RNA and protein are likely to interact. Using this threshold, accuracies of the classifiers ranged from 87 - 90% in cross-validation evaluation experiments on benchmark datasets. When classifiers were tested on independent (blind) datasets of RPIs, accuracies of the classifiers ranged from 57 – 99%.

DNMT3B

Interaction probabilities **BncRNA_complete_sequence + dnmt3b_protein**

Prediction using RF classifier **0.8**

Prediction using SVM classifier **0.98**

What do these probabilities mean?

Interaction probabilities generated by RPISeq range from 0 to 1. In performance evaluation experiments, predictions with probabilities > 0.5 were considered “positive,” i.e., indicating that the corresponding RNA and protein are likely to interact. Using this threshold, accuracies of the classifiers ranged from 87 - 90% in cross-validation evaluation experiments on benchmark datasets. When classifiers were tested on independent (blind) datasets of RPIs, accuracies of the classifiers ranged from 57 – 99%.

Interaction probabilities **BncRNA_subregion2 + dnmt3b_protein**

Prediction using RF classifier **0.65**

Prediction using SVM classifier **0.91**

What do these probabilities mean?

Interaction probabilities generated by RPISeq range from 0 to 1. In performance evaluation experiments, predictions with probabilities > 0.5 were considered “positive,” i.e., indicating that the corresponding RNA and protein are likely to interact. Using this threshold, accuracies of the classifiers ranged from 87 - 90% in cross-validation evaluation experiments on benchmark datasets. When classifiers were tested on independent (blind) datasets of RPIs, accuracies of the classifiers ranged from 57 – 99%.

*RPI-Pred software*

**DNMT1**

**BncRNA_complete_sequence + dnmt1_protein**


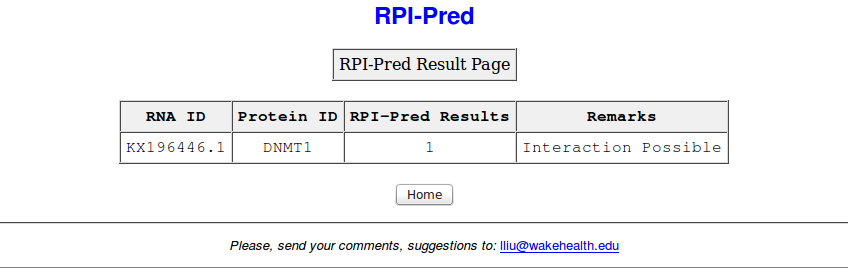


**BncRNA_subregion2 + dnmt1_protein**


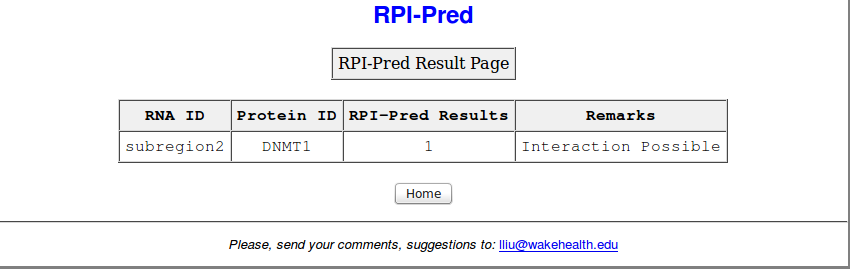


**DNMT3A**

**BncRNA_complete_sequence + dnmt3a_protein**


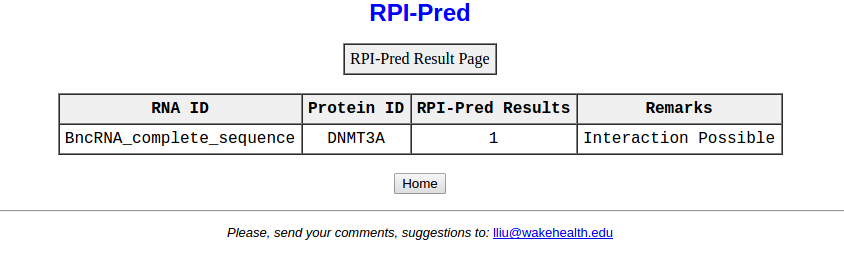


**BncRNA_subregion2 + dnmt3a_protein**


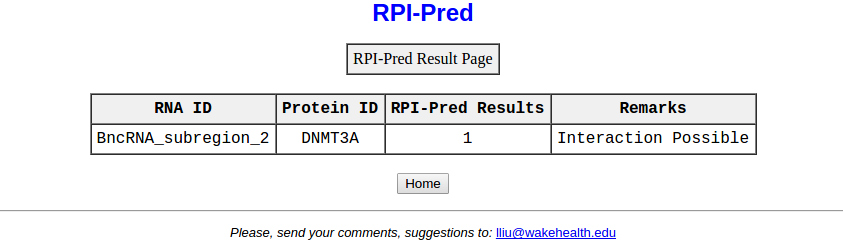


**DNMT3B**

**BncRNA_complete_sequence + dnmt3b_protein**


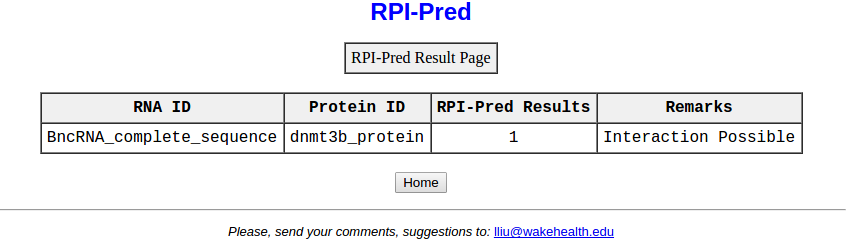


**BncRNA_subregion2 + dnmt3b_protein**


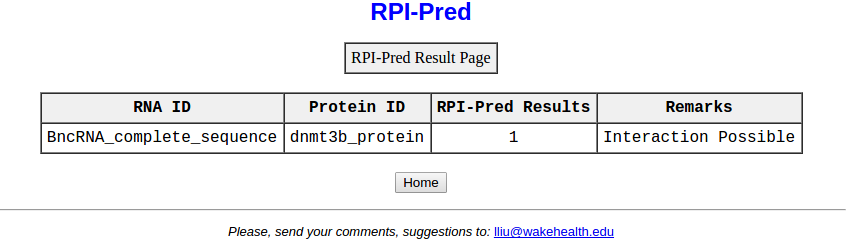

Supplement: Supplementary file 1 [file Data_Sheet_1.docx]
